# Supplementary material for: Spatiotemporal Control of ULK1 Activation by NDP52 and TBK1 during Selective Autophagy
Source: Mol Cell. 2019 Apr 18;74(2):347–362.e6. doi: 10.1016/j.molcel.2019.02.010 (PMC6642318; doi:10.1016/j.molcel.2019.02.010)

Molecular Cell, Volume *74*

Supplemental Information

Spatiotemporal Control of ULK1 Activation by NDP52 and TBK1 during Selective Autophagy

Jose Norberto S. Vargas, Chunxin Wang, Eric Bunker, Ling Hao, Dragan Maric, Giampietro Schiavo, Felix Randow, and Richard J. Youle

SUPPLEMENTAL FIGURES

Figure S1. NDP52 interacts with ULK1 complex through FIP200, related to Figures 1, 2 and 3.

(A) Western analysis of GFP-TRAP from HeLa cells stably expressing mCherry-Parkin and GFP-NDP52. Cells were treated with OA and Bafilomycin (100 nM) for 2 h. (B) Western analysis of NDP52 levels in WT or FIP200 KO HeLa cells.

(C) Endogenous immunoprecipitation of ULK1 in WT HeLa or FIP200 KO cells. (D) Western analysis of GFP-TRAP from HEK293T cells transiently expressing GFP-ULK1, FLAG-NDP52, and HA-FIP200. (E) Confocal imaging of HeLa cells stably expressing mito-mKeima, FRB-Fis1 and FKBP-GFP-NDP52 WT or ΔSKICH mutant. Cells were transfected with HA-FIP200, followed by 24 h treatment of Rapalog. Cells were then immunostained for HA and Tom20. Arrows indicate triple colocalization of FKBP-GFP-NDP52, FIP200, and Tom20. Scale bars: 10 μm

Figure S2. Mitochondrial recruitment of NDP52 in the absence of TBK1, related to Figures 4 and 6.

(A) Confocal images of WT or TBK1 KO HeLa cells stably expressing BFP-NDP52 and mCherry-Parkin. Cells were treated with OA and Bafilomycin (200 nM) for 3 h. (B-C) Quantification of Pearson's correlation coefficient between NDP52 and Parkin with or

without OA treatment for 3 or 24 h. (D) Confocal imaging of WT HeLa cells expressing various BFP-NDP52 mutants after 3 h treatment with OA (See Fig. 3A). (E) Western analysis of HA-Ub immunoprecipitation. HEK293T cells transiently expressing HA-Ubiquitin with FLAG-NDP52, FLAG-NDP52-ΔSKICH-ΔCLIR, or Flag-NDP52 (C425A). Red arrows indicate NDP52 that is bound to ubiquitin. Data are represented as mean ± SD. p-value: * = < 0.05; ** < 0.01; *** < 0.001; **** < 0.00001; ns., not significant. Scale bars: 10 μm

Figure S3. Ectopic mitochondrial localization of ATG16L1 FIP200-binding domain can initiate mitophagy via FIP200, related to Figures 5, 6 and 7.

(A) Diagram of FIP200 binding domain (FBD) from ATG16L1 fused with FKBP. (B) Quantification of FACS analysis for mito-mKeima. Cells stably expressing mito-mKeima, FRB-Fis1, and FKBP-GFP-FBD or FKBP-GFP-FBD (E230R/E241R) mutant were treated with Rapalog for 24 h. (C) Western analysis of GFP-TRAP assay performed on HEK293T cells overexpressing FKBP-GFP-FBD and (E230R/E241R) mutant. (D) Quantification of FACS ratiometric analysis of mito-mKeima. Cells stably expressing mito-mKeima, FRB-Fis1, and FKBP-GFP-FBD were treated with Rapalog alone or with co-treatment of ULK1/2 inhibitor (MRT68921 1 μM) or TBK1 inhibitor (MRT67307 2 μM) for 24 h. (E) Quantification of FACS ratiometric analysis of mito-mKeima. WT or AMPK alpha 1/2 double KO (DKO) stably expressing mito-mKeima, FRB-Fis1, and FKBP-GFP-FBD. Cells were treated with Rapalog for 24 h. All FACS quantifications: n = 3

biological replicates. Data are represented as mean ± SD. p-value: * = < 0.05; ** < 0.01; *** < 0.001; **** < 0.00001; ns., not significant.

Figure S4. LC3 lipidation defect in the absence of AMPK in human cells and effect of ULK1 autophosphorylation sites, related to Figure 5.

(A) Western analysis of WT or FIP200 KO HeLa cells after starvation with HBSS with or without Bafilomycin. (B-C) Western analysis. WT U2OS and AMPK DKO were starved for 4 h (B) or 24h (C) with or without Bafilomycin. (D) Cells in (B) were treated with Torin1 for 4h with or without Bafilomycin. (E) FACS ratiometric mito-mKeima analysis. HeLa cells expressing FKBP-GFP-ULK1 WT, S1042A, or S1047A, with FRB-Fis1, and mito-mKeima were treated with Rapalog for 24 h. All FACS quantifications: n = 3 biological replicates. Data are represented as mean ± SD. p-value: * = < 0.05; ** < 0.01; *** < 0.001; **** < 0.00001; ns., not significant

Figure S5. TBK1 positively regulates mitophagy rate with NDP52, related to Figure 6

(A) Western analysis of COXII and Tim23 to measure mitophagy in WT or TBK1 KO HeLa cells. Cells stably expressing mCherry-Parkin were treated with OA for 6, 18, or 24 h. (B) Western analysis of mitophagy in WT or TBK1 KO HCT116 cells. Cells stably expressing mCherry-Parkin were treated with OA for 6, 18 and 24 h. (C) Western

analysis of HCT116 after treatment of OA with or without Bafilomycin for 6 or 16h. (D) Western analysis of ULK1 complex levels in WT or TBK1 KO HeLa cells. (E) Schematics of chimeric NDP52-TBK1 generated for mitophagy rescue analysis in (F) and (G). (F-G) FACS ratiometric analysis of mito-mKeima in (F) TBK1 KO cells or in (G) OPTN, NDP52, Tax1bp1 TKO cells expressing the various TBK1-NDP52 constructs. Cells were treated with OA for 4 h prior to FACS. All FACS quantifications: n = 3 biological replicates. Data are represented as mean ± SD. p-value: * = < 0.05; ** < 0.01; *** < 0.001; **** < 0.00001; ns., not significant

Figure S6. Autophosphorylation of TBK1 upon cargo localization, related to Figure 6.

(A-B) Confocal imaging of phosphorylated TBK1 S172. HeLa cells expressing FRB-Fis1, mito-mKeima, and FKBP-GFP-TBK1 (A) or K38M kinase-dead FKBP-GFP-TBK1 (B) were treated with Rapalog and QVD for 24h. Cells were then immunostained for Tom20 and TBK1 S172 prior to imaging. (C-D) Pearson's correlation coefficient between GFP-FKBP-TBK1 or K38M mutant and Tom20 (C) and between TBK1 phosphorylated at S172 and Tom20. (E) Western analysis of S172 phosphorylation. HeLa cells expressing FRB-Fis1, mito-mKeima, and FKBP-GFP-TBK1 or or K38M kinase-dead FKBP-GFP-TBK1 were treated with Rapalog for 24 h. Data are represented as mean ± SD. p-value: * = < 0.05; ** < 0.01; *** < 0.001; **** < 0.00001; ns., not significant

Figure S7. Pexophagy induced by NDP52, ULK1, and TBK1 localization, related to Figure 5, 6, and 7.

(A) Confocal imaging of catalase staining in WT or 6KO (LC3/GABARAP hexa KO) HeLa cells. Cells are transiently expressing FKBP-FLAG/HA-NDP52, mito-GFP, and FRB-PMP34 and treated with Rapalog for 24 h. Cells were then fixed and stained for catalase and HA to ensure expression of FKBP construct (not shown). White asterisks denote cytosolic catalase staining while blue asterisks show aggregated catalase staining. (B) Quantification of catalase staining morphology in (A). (C) Quantification of peroxisome morphology in WT, 5KO (OPTN, NDP52, Tax1bp1, p62, NBR1 penta KO), and 6KO cells. Cells are transiently expressing FKBP/FLAG-HA-ULK1, mito-GFP, and FRB-PMP34. (D) Confocal imaging of catalase staining in WT or 5KO HeLa cells transiently expressing FKBP-FLAG/HA-TBK1, mito-GFP, and FRB-PMP34. Cells were treated with Rapalog for 24 h, fixed, then stained for catalase and HA (not shown). (E) Quantification of cells with either normal, cytosolic, or aggregated catalase staining in (D). (F) Quantification of catalase staining morphology in cells expressing FLAG/HA-NDP52 in WT or FIP200 KO cells. For all imaging experiments: Mito-GFP is used to track transfected cells, n = 3 biological replicates with at least 150 cells per condition counted in each experiment. Data are represented as mean ± SD. p-value: * = < 0.05; ** < 0.01; *** < 0.001; **** < 0.00001; ns., not significant.

Fig S1. NDP52 interacts with ULK1 complex through FIP200

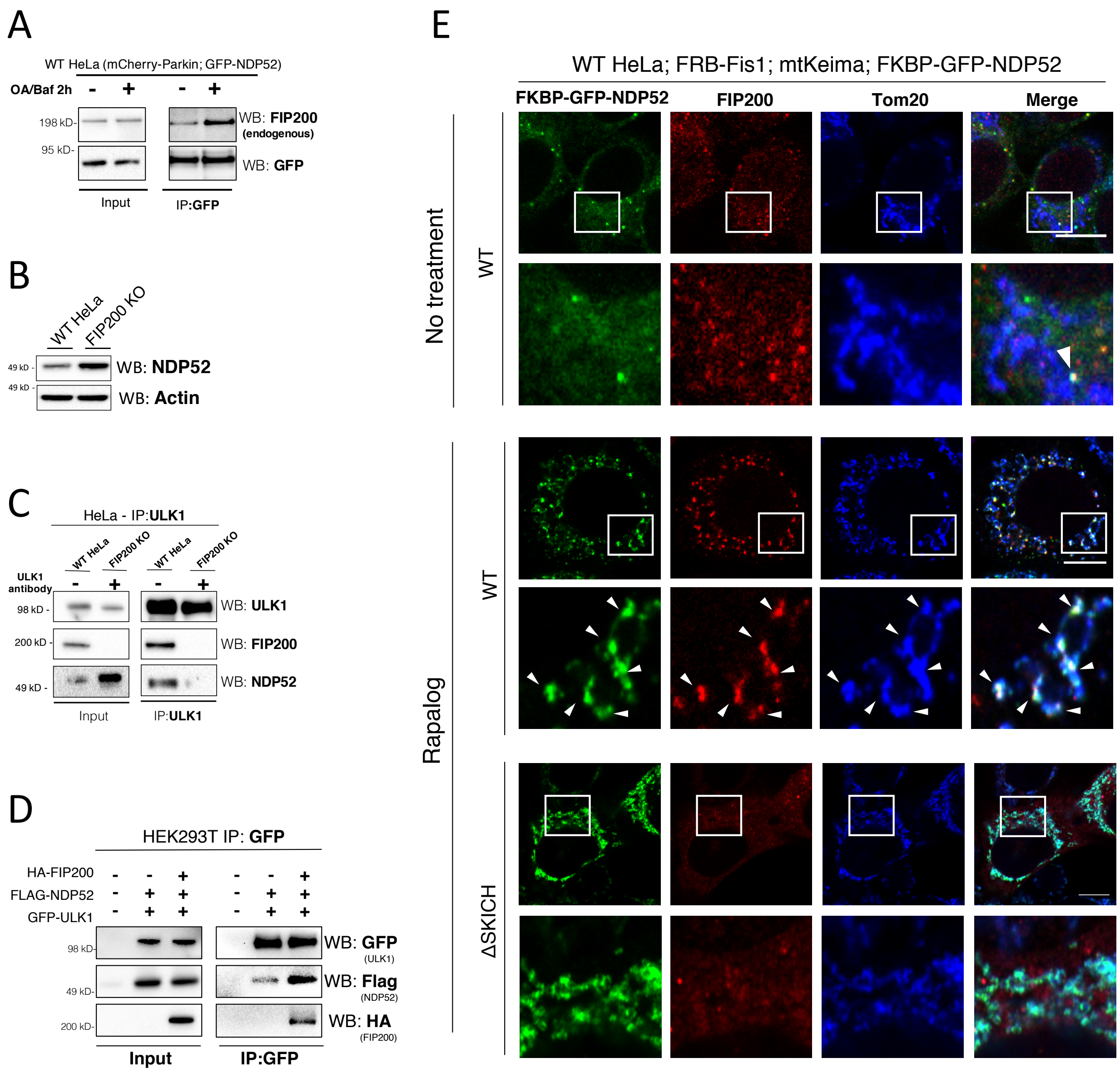

Fig S2. Mitochondrial recruitment of NDP52 in the absence of TBK1

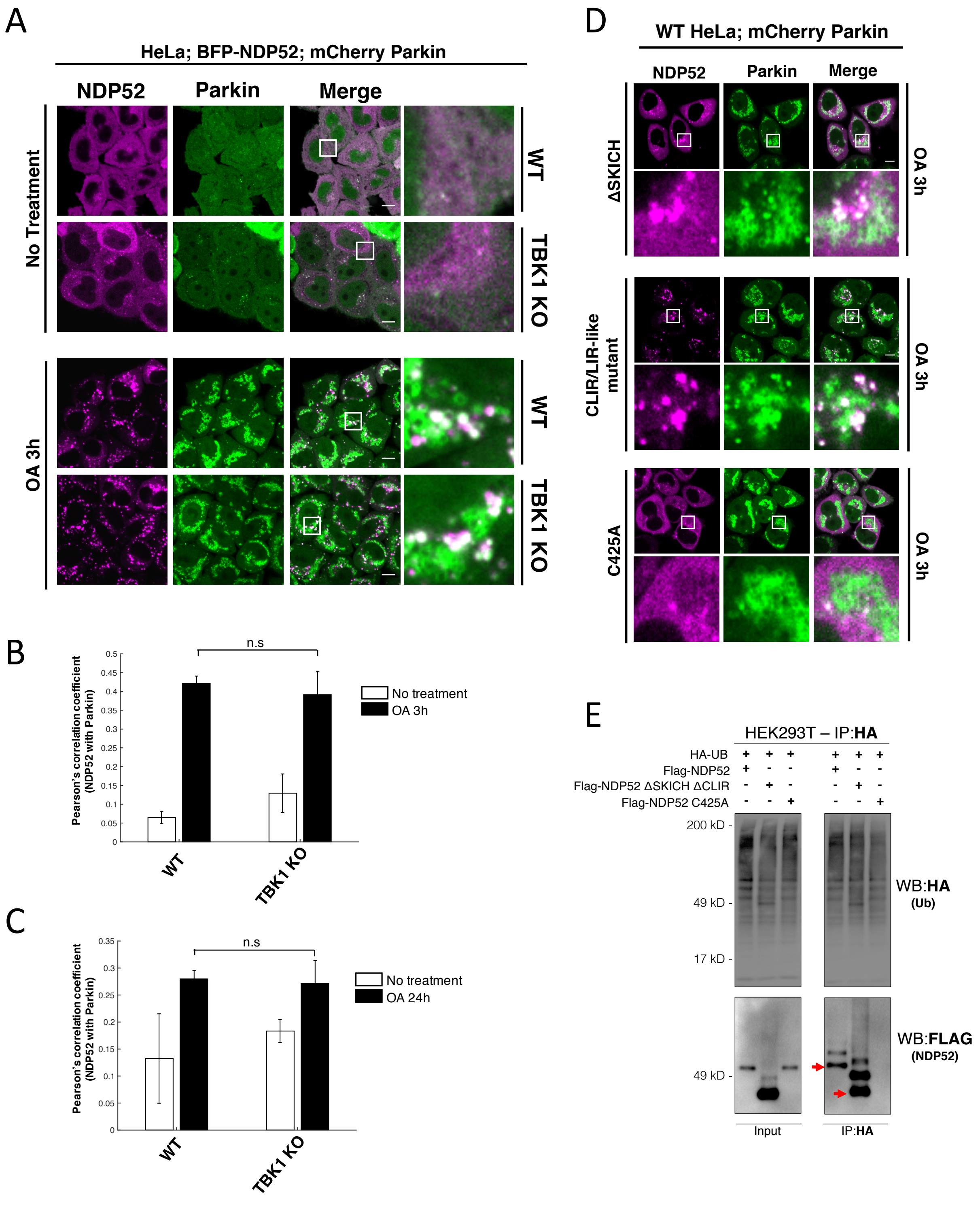

Fig. S3 Ectopic mitochondrial localization of ATG16L1 FBD domain can initiate mitophagy via FIP200

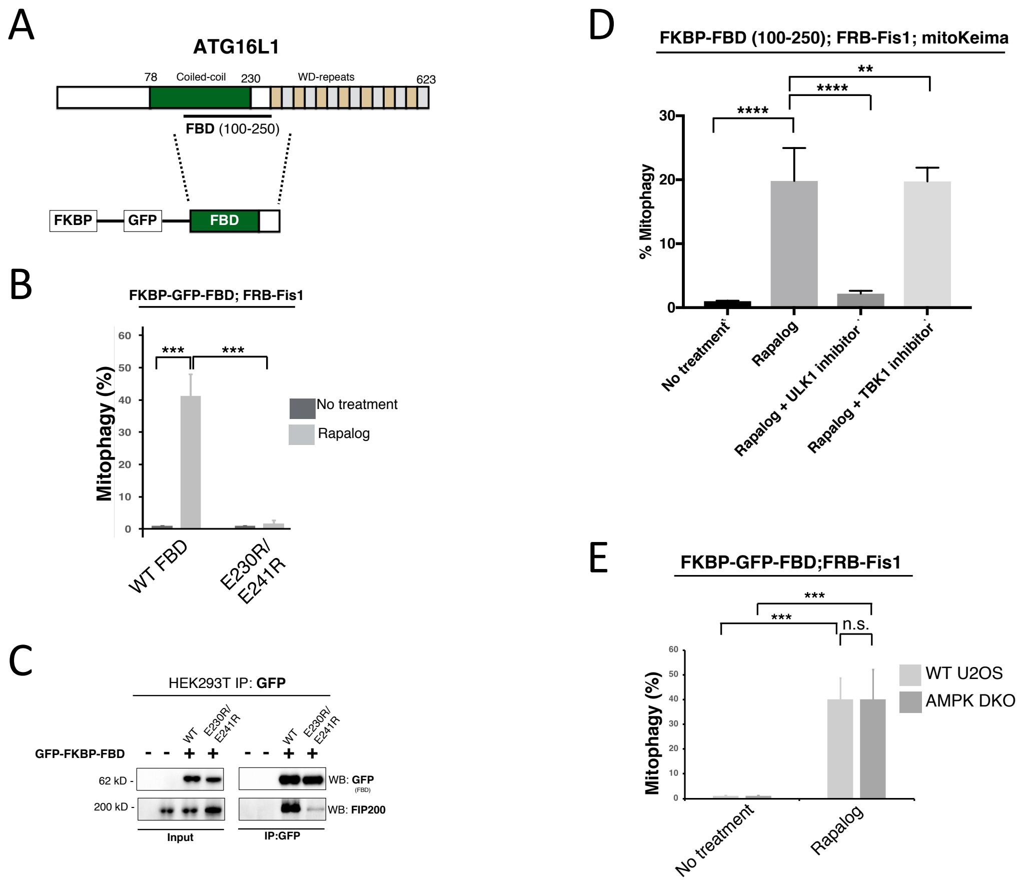

Fig. S4 LC3 lipidation defect in the absence of AMPK in human cells and effect of ULK1 autophosphorylation sites

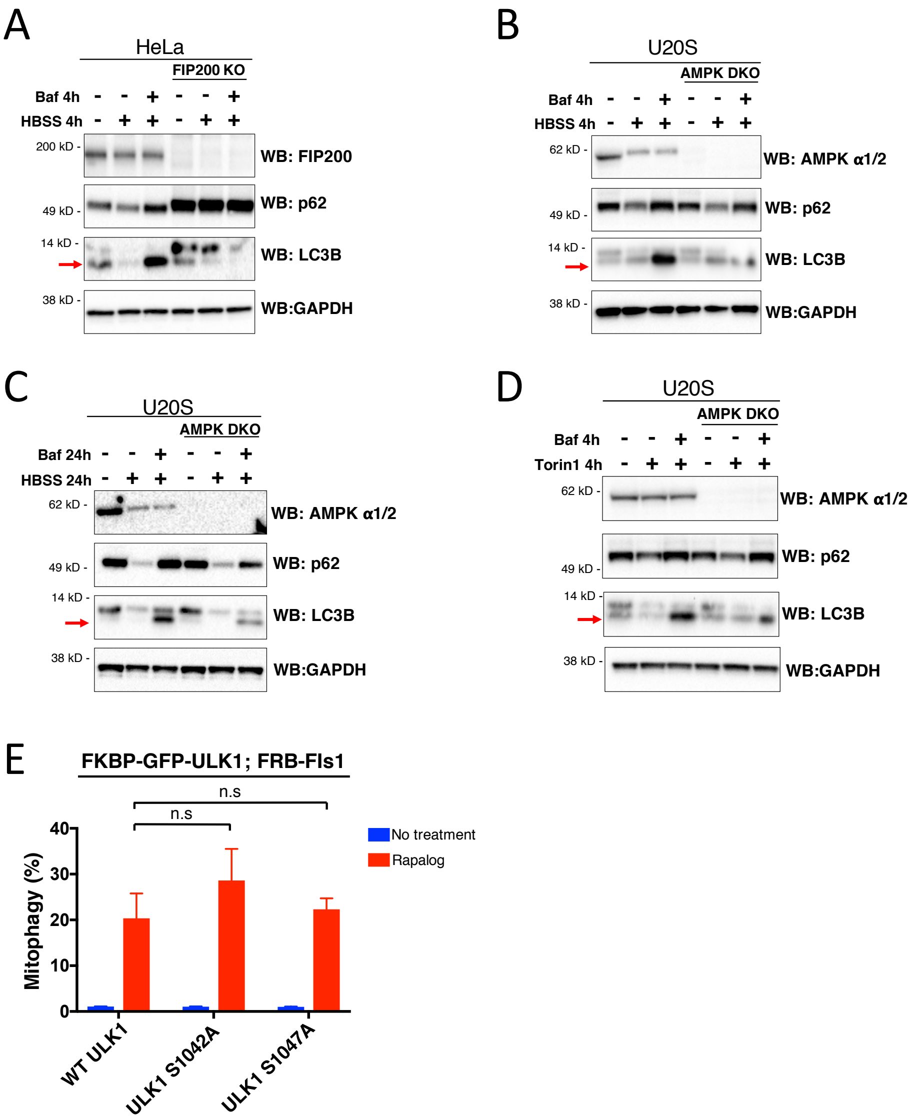

Fig. S5 TBK1 functions with NDP52 to positively regulate mitophagy rate

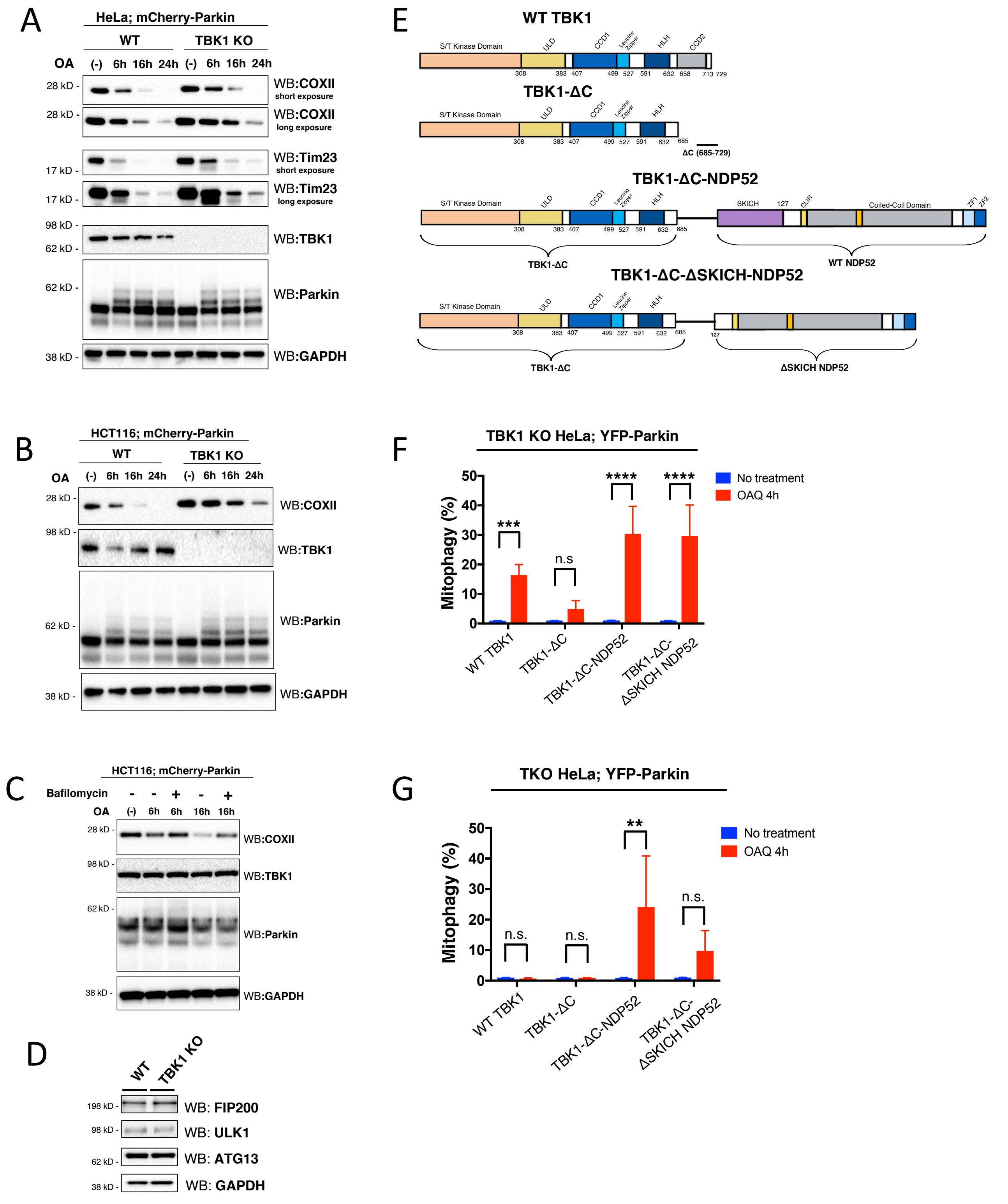

Fig. S6 TBK1 autophosphorylation on cargo

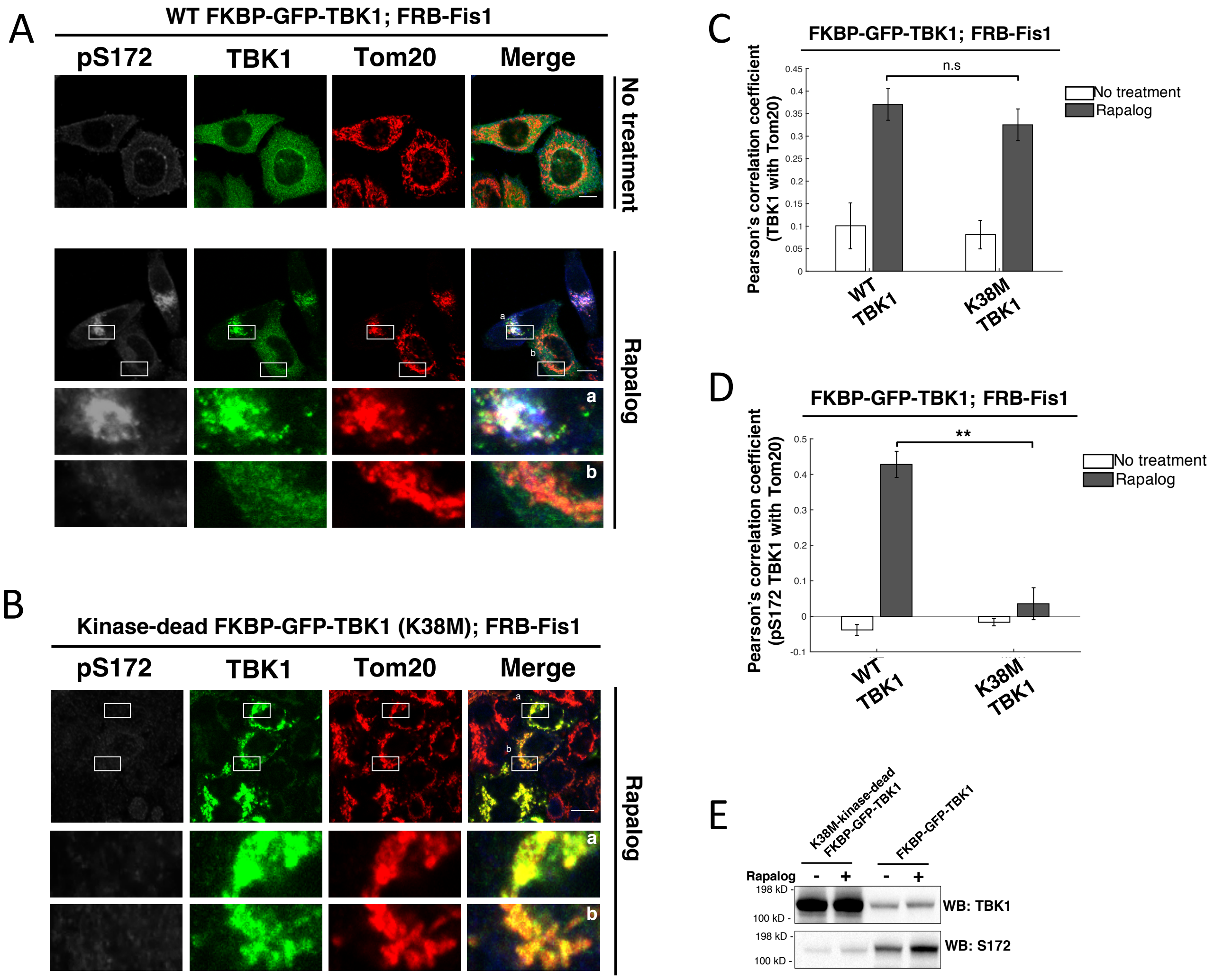

Fig S7 Pexophagy induced by NDP52, ULK1 and TBK1 localization

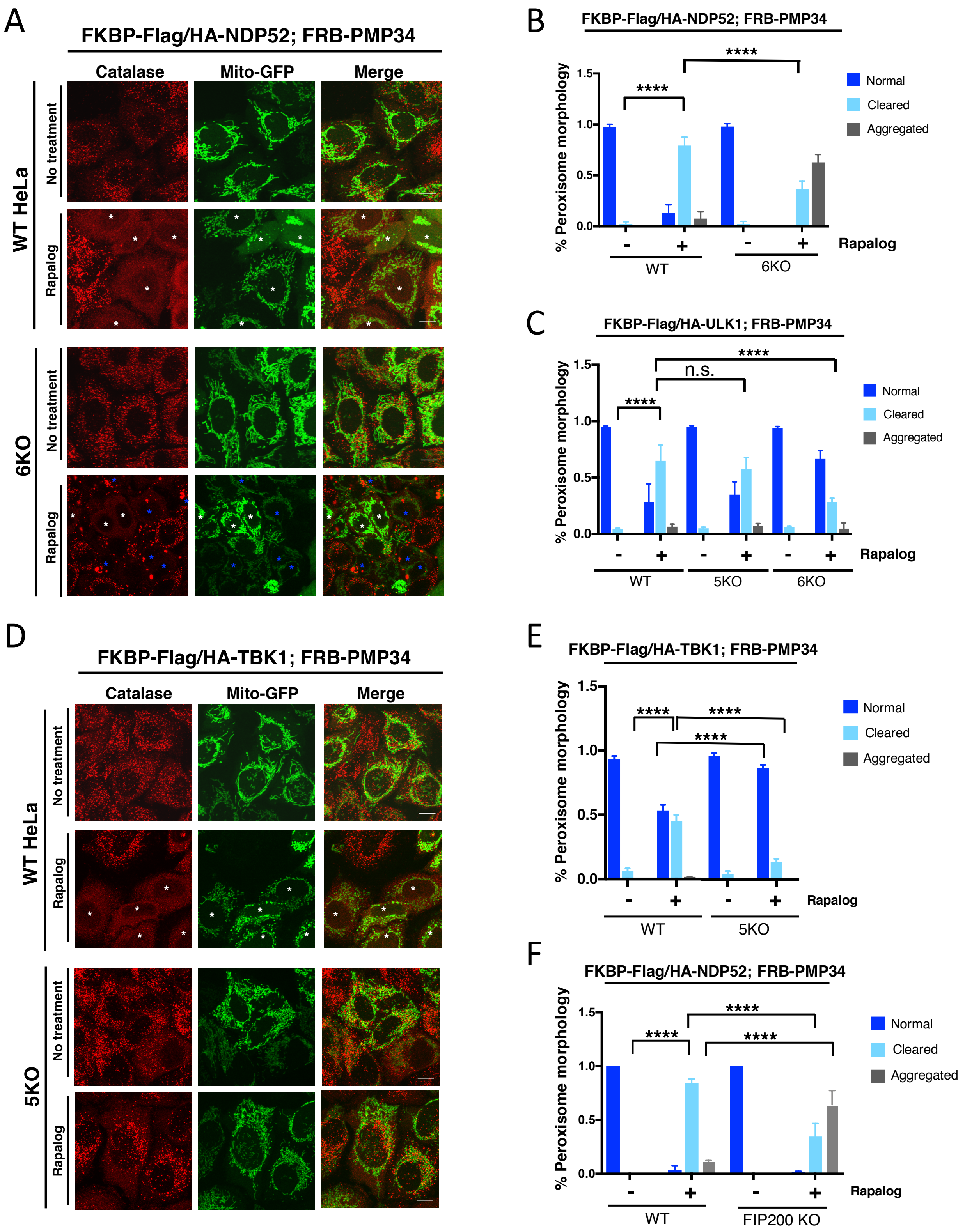

Supplement: Document S1. Figures S1–S7 [file mmc1.pdf]
