# Supplementary material for: Spatiotemporal Control of ULK1 Activation by NDP52 and TBK1 during Selective Autophagy
Source: Mol Cell. 2019 Apr 18;74(2):347–362.e6. doi: 10.1016/j.molcel.2019.02.010 (PMC6642318; doi:10.1016/j.molcel.2019.02.010)

Molecular Cell

Article

Spatiotemporal Control of ULK1 Activation by NDP52 and TBK1 during Selective Autophagy

Graphical Abstract

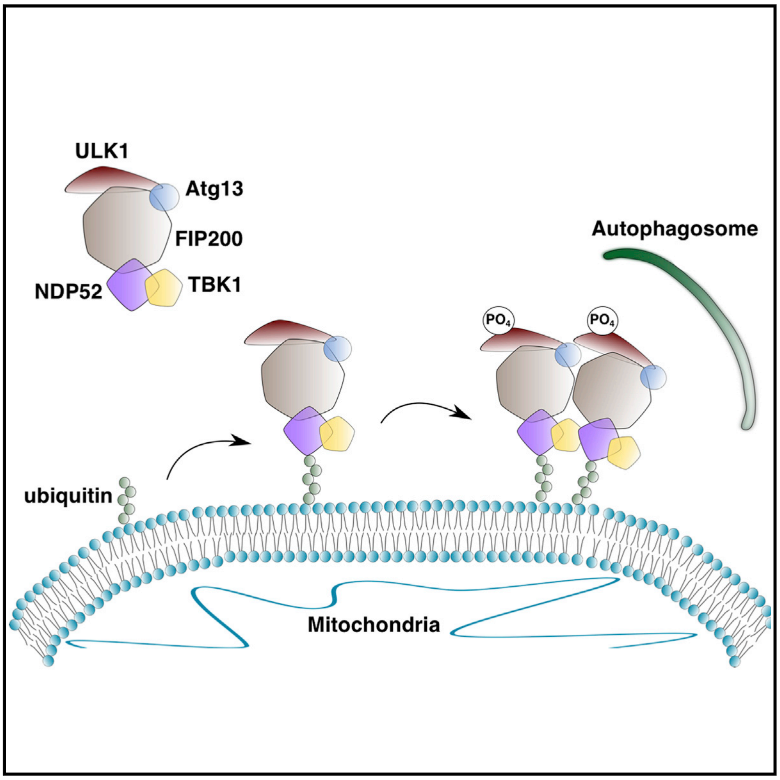

Highlights

- NDP52 associates with the ULK1 complex through FIP200, facilitated by TBK1
- NDP52/TBK1 targets ULK1 to cargo to initiate autophagy in the absence of LC3
- ULK1 is activated on cargo independently of AMPK and mTOR activity
- Ectopic recruitment of FIP200-binding peptide is sufficient to degrade cargo

Authors

Jose Norberto S. Vargas,
Chunxin Wang, Eric Bunker, ...,
Giampietro Schiavo, Felix Randow,
Richard J. Youle

Correspondence

youler@ninds.nih.gov

In Brief

The targeting and activation mechanism of ULK1 complex during selective autophagy has been unclear. Here, Vargas et al. show that NDP52/TBK1 target the ULK1 complex to cargo in an LC3-independent manner and that ULK1 kinase activation is coupled to cargo localization, circumventing energy-sensing pathways.

Vargas et al., 2019, Molecular Cell *74*, 347–362
April 18, 2019 © 2019 MRC Laboratory of Molecular Biology.
Published by Elsevier Inc.
https://doi.org/10.1016/j.molcel.2019.02.010

CellPress

Spatiotemporal Control of ULK1 Activation by NDP52 and TBK1 during Selective Autophagy

Jose Norberto S. Vargas,[1,4,8] Chunxin Wang,[1,8] Eric Bunker,[1] Ling Hao,[1] Dragan Maric,[5] Giampietro Schiavo,[4,6,7] Felix Randow,[2,3] and Richard J. Youle[1,9,*]
[1]Biochemistry Section, Surgical Neurology Branch, National Institute of Neurological Disorders and Stroke, National Institutes of Health, Bethesda, MD 20892, USA
[2]MRC Laboratory of Molecular Biology, Division of Protein and Nucleic Acid Chemistry, Francis Crick Avenue, Cambridge CB2 0QH, UK
[3]University of Cambridge, Department of Medicine, Addenbrooke's Hospital, Cambridge CB2 0QQ, UK
[4]Department of Neuromuscular Disorders, UCL Institute of Neurology, University College London, London WC1N 3BG, UK
[5]Flow and Imaging Cytometry Core Facility, National Institute of Neurological Diseases and Stroke, Bethesda, MD 20892, USA
[6]UK Dementia Research Institute at UCL, UCL Queen Square Institute of Neurology, University College London, London WC1E 6BT, UK
[7]Discoveries Centre for Regenerative and Precision Medicine, UCL Campus, University College London, London WC1E 6BT, UK
[8]These authors contributed equally
[9]Lead Contact
*Correspondence: youler@ninds.nih.gov
https://doi.org/10.1016/j.molcel.2019.02.010

SUMMARY

Selective autophagy recycles damaged organelles and clears intracellular pathogens to prevent their aberrant accumulation. How ULK1 kinase is targeted and activated during selective autophagic events remains to be elucidated. In this study, we used chemically inducible dimerization (CID) assays in tandem with CRISPR KO lines to systematically analyze the molecular basis of selective autophagosome biogenesis. We demonstrate that ectopic placement of NDP52 on mitochondria or peroxisomes is sufficient to initiate selective autophagy by focally localizing and activating the ULK1 complex. The capability of NDP52 to induce mitophagy is dependent on its interaction with the FIP200/ULK1 complex, which is facilitated by TBK1. Ectopically tethering ULK1 to cargo bypasses the requirement for autophagy receptors and TBK1. Focal activation of ULK1 occurs independently of AMPK and mTOR. Our findings provide a parsimonious model of selective autophagy, which highlights the coordination of ULK1 complex localization by autophagy receptors and TBK1 as principal drivers of targeted autophagosome biogenesis.

INTRODUCTION

Autophagy is critical for the bulk degradation of various intracellular components and for cellular homeostasis (Dikic and Elazar, 2018; Mizushima et al., 2011). Moreover, autophagosomes can be built around specific subcellular components destined for selective degradation (Gatica et al., 2018). Selective autophagic targets, such as damaged mitochondria or invading bacteria, first become tagged by ubiquitin, which serves as an "eat me" signal. Then autophagy receptors bind ubiquitinated cargo via their ubiquitin binding domains (Randow and Youle, 2014; Behrends and Fulda, 2012; Kirkin et al., 2009). Autophagy-related (ATG) proteins then assemble to facilitate the formation of early double-membrane structures called isolation membranes that expand around the cargo. Once fully encapsulating cargo, the autophagosome fuses with lysosomes for content degradation (Tooze, 2013; Bento et al., 2016). The orchestrated steps of autophagosome formation, involving many ATG proteins, have been elucidated (Behrends et al., 2010; Itakura and Mizushima, 2010). However, there is no clear mechanism known to link cargo recognition with autophagosome biogenesis in mammals, and it is uncertain what provides the targeting mechanism to nucleate the early autophagy machinery in the vicinity of organelles destined for degradation.

The prevailing model for selective autophagy posits that autophagy receptors, such as NDP52, bridge ubiquitinated cargos through their ubiquitin binding domains with preformed, expanding autophagosomes studded with lipidated LC3 via their LC3-interacting regions (Kirkin et al., 2009; Birgisdottir et al., 2013). However, recent studies exploring the role of LC3/GABARAP family proteins during mitophagy found that autophagosomes can still selectively form around cargo in the absence of all LC3/GABARAP proteins (Nguyen et al., 2016; Pontano Vaites et al., 2017). Instead, these studies found that LC3/GABARAP proteins are important for lysosomal fusion steps. Consistently, early ATG proteins are recruited to mitochondria during CCCP-induced mitophagy even in cells lacking ATG3, an E2-like enzyme critical for LC3-PE conjugation (Itakura et al., 2012). Thus, the function of autophagy receptors in relation to LC3 and autophagosome recruitment to cargo is unclear. We previously demonstrated that CRISPR KO cell lines lacking all known sequestosome-like autophagy receptors have a defect in ULK1 complex recruitment and mitophagy (Lazarou et al., 2015), which suggests a role for autophagy receptors in initiating this first step in isolation membrane formation directly on the cargo. Therefore, autophagy receptors, such as NDP52, OPTN, and Tax1bp1, could potentially be the missing link

This is an open access article under the CC BY license (http://creativecommons.org/licenses/by/4.0/).

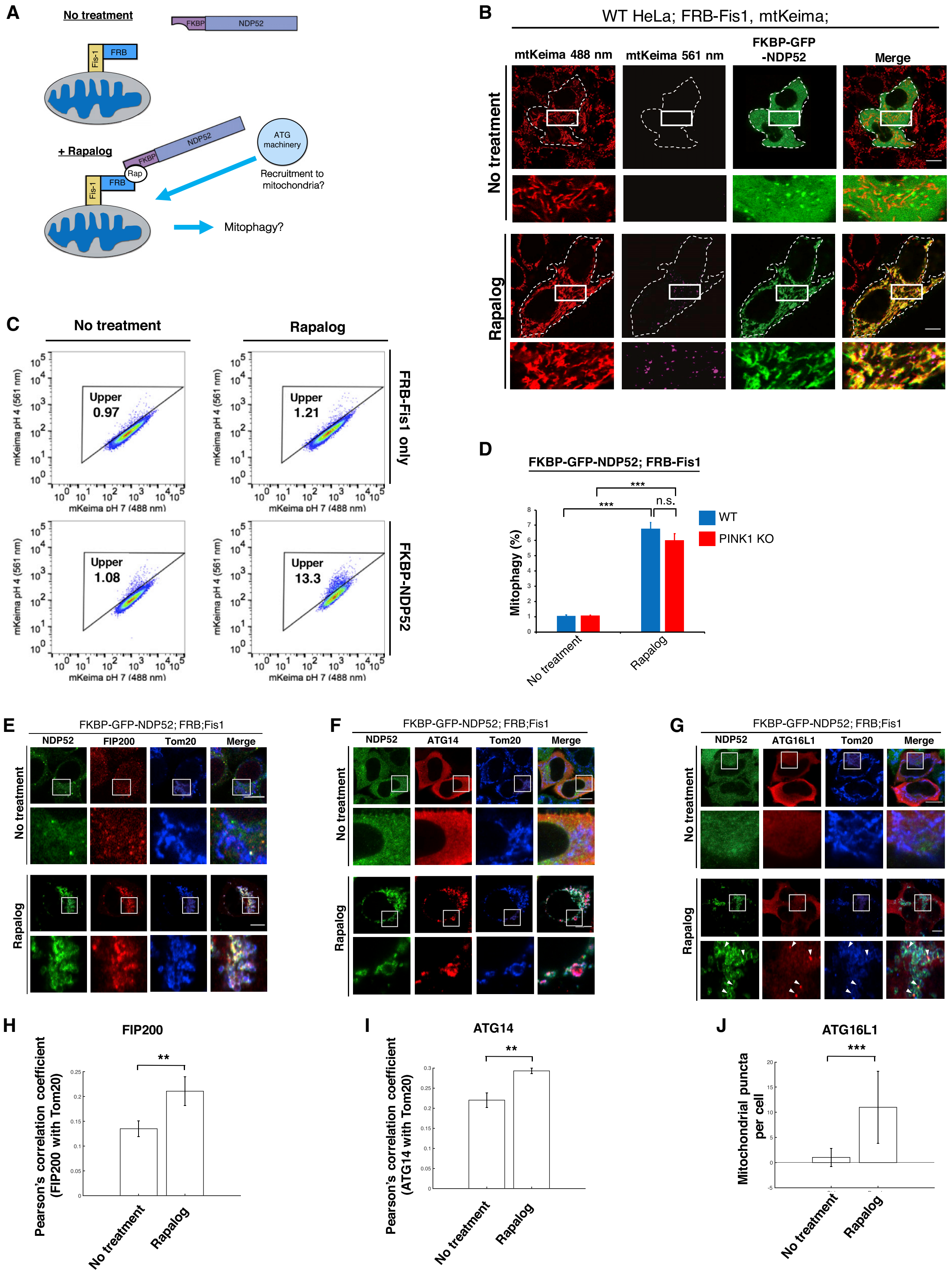

(legend on next page)

between cargo recognition and the spatiotemporal initiation of autophagosome biogenesis.

The ULK1 complex, the most upstream machinery during autophagosome biogenesis, consists of ULK1 kinase, FIP200, ATG13, and ATG101 (Mizushima et al., 2011). During starvation-induced non-selective autophagy, AMPK kinase is activated by loss of nutrient availability, which in turn activates ULK1 to initiate clearance of intracellular contents (Kim et al., 2011; Garcia and Shaw, 2017). Although AMPK activation has been linked to mitochondrial dynamics (Toyama et al., 2016) and to mitochondrial biogenesis (Jäger et al., 2007; Cantó et al., 2009), it is less clear whether AMPK is involved in PINK1/Parkin-dependent mitophagy or other modes of selective autophagy (Herzig and Shaw, 2018). Conversely, when cells are nutrient replete, mTOR kinase phosphorylates ULK1 to constitutively inhibit autophagy (Kim et al., 2011). How ULK1 overcomes mTOR inhibition during fed states to initiate selective degradation of various cargo is unknown. Studies on Atg1, the yeast homolog of ULK1, demonstrated that Atg1 autoactivates on cargo by local clustering (Torggler et al., 2016; Kamber et al., 2015). Researchers investigating ULK1 activation induce autophagy by globally depleting cellular energy and nutrient availability (Alers et al., 2012; Russell et al., 2014). Such widespread alteration in energetic states leading to ULK1 activation may not recapitulate the function of ULK1 during selective autophagy (Papinski and Kraft, 2016), during which bioenergetic states can be normal. Thus, it is unclear how ULK1 is activated during selective autophagy.

Another multifunctional kinase implicated in selective autophagy is TANK-Binding Kinase1 (TBK1). Recent reports have shown that TBK1 phosphorylates autophagy receptors, which affects their affinity toward ubiquitin chains on damaged cargo, as well as LC3-II (Wild et al., 2011; Moore and Holzbaur, 2016; Heo et al., 2015; Richter et al., 2016). Furthermore, TBK1 aids in the initiation of bacterial autophagy (Thurston et al., 2016) and mediates signaling crosstalk between energy-sensing and inflammatory pathways through AMPK (Zhao et al., 2018), indicating that TBK1 controls multiple aspects of autophagy.

In this study, we delineate the mechanism for the initiation of selective autophagy. We find that NDP52 and TBK1 cooperate to recruit the ULK1 complex to ubiquitinated cargo, leading to ULK1 kinase activation, which occurs independently of energy-sensing pathways.

RESULTS

NDP52-Mediated Mitophagy by Chemically Inducible Protein Localization

During depolarization-induced mitophagy, NDP52 and a number of other autophagy-related proteins are recruited to the mitochondria. To isolate the role of NDP52 in autophagosome biogenesis, we used chemically induced dimerization (CID) assays to directly recruit NDP52 to mitochondria without perturbing cell metabolism (Figure 1A). We stably expressed FKBP-GFP-NDP52 and FRB-Fis1 in cells also expressing mito-mKeima to quantify mitophagic flux (Lazarou et al., 2015; Sun et al., 2017). Localizing NDP52 to the mitochondria with a compound that induces the dimerization of FKBP and FRB results in the appearance of mitolysosomes detected by imaging (Figure 1B) and by FACS analysis (Figure 1C). The mitolysosomes observed are not due to Parkin-mediated mitophagy, since HeLa cells do not express endogenous Parkin (Burman et al., 2017). We also localized NDP52 to mitochondria in PINK1 KO cells and found no difference from WT cells in mitolysosome formation (Figure 1D). Thus, ectopic recruitment of NDP52 can induce mitophagy independently or downstream of the PINK1/Parkin pathway. Consistently, forced mitochondrial localization of NDP52 results in the recruitment of the ATG proteins: FIP200, ATG14, and ATG16L1 (Figures 1E–1G; quantified in Figures 1H–1J). ATG14 and ATG16L1 are downstream of FIP200 and involved in phagophore maturation (Wesselborg and Stork, 2015). Together, these results indicate that targeting of NDP52 to cargo is sufficient to mobilize ATG proteins to initiate selective cargo degradation.

NDP52 Interacts with the ULK1 Complex through FIP200

That NDP52 can directly initiate mitophagy and recruit various ATG proteins when experimentally localized to mitochondria suggests that NDP52 may be interacting with the autophagy machinery. Consistent with this notion, a previous unbiased yeast two-hybrid screen revealed an interaction between FIP200 and NDP52 (Wang et al., 2011). Immunoprecipitation experiments with either ULK1 or FIP200 revealed that endogenous NDP52 associates with the endogenous ULK1 complex: ULK1 kinase, ATG13, and FIP200 (Figures 2A and 2B). Moreover, the association of NDP52 to the ULK1 complex is enhanced during

Figure 1. Ectopic Recruitment of NDP52 to Mitochondria Can Initiate Mitophagy by Recruiting ATG Machinery

(A) Schematic of chemically inducible dimerization assay to control mitochondrial localization of NDP52.

(B) Live confocal imaging of HeLa cells stably expressing mito-mKeima and FRB-Fis1 and transiently expressing FKBP-GFP-NDP52. Cells were treated with Rapalog for 24 h. Cytosolic mitochondria (488 nm; red) and mitolysosomes (561 nm; purple) are shown.

(C) FACS plots showing mito-mKeima ratio (561/488 nm). HeLa cells stably expressing mito-mKeima, FRB-Fis1, and FKBP-GFP-NDP52 were treated with Rapalog for 24 h.

(D) Quantification of mito-mKeima ratiometric FACS analysis of WT or PINK1 KO HeLa cells stably expressing mito-mKeima, FRB-Fis1, and FKBP-GFP-NDP52 after 24 h of Rapalog treatment. n = 3 biological replicates.

(E–G) Confocal images of HeLa cells stably expressing mito-mKeima, FRB-Fis1, and FKBP-GFP-NDP52. Cells were transfected with (E) HA-FIP200, (F) HA-ATG14, and (G) Flag-ATG16L1. Cells were then treated with Rapalog for 24 h, fixed, then immunostained for Tom20 with either HA or Flag.

(H) Quantification of FIP200 recruitment to mitochondria by Pearson's correlation coefficient as in (E).

(I) Quantification of ATG14 recruitment to mitochondria by Pearson's correlation coefficient as in (F).

(J) Quantification of the number of ATG16L1 foci on mitochondria before and after Rapalog treatment as in (G). Arrows indicate foci of ATG16L1 colocalized with Tom20.

All FACS quantifications: n = 3. Data are represented as mean ± SD. p value: * = < 0.05; ** < 0.01; *** < 0.001; **** < 0.00001; ns., not significant. Scale bars: 10 μm. See also Figure S1.

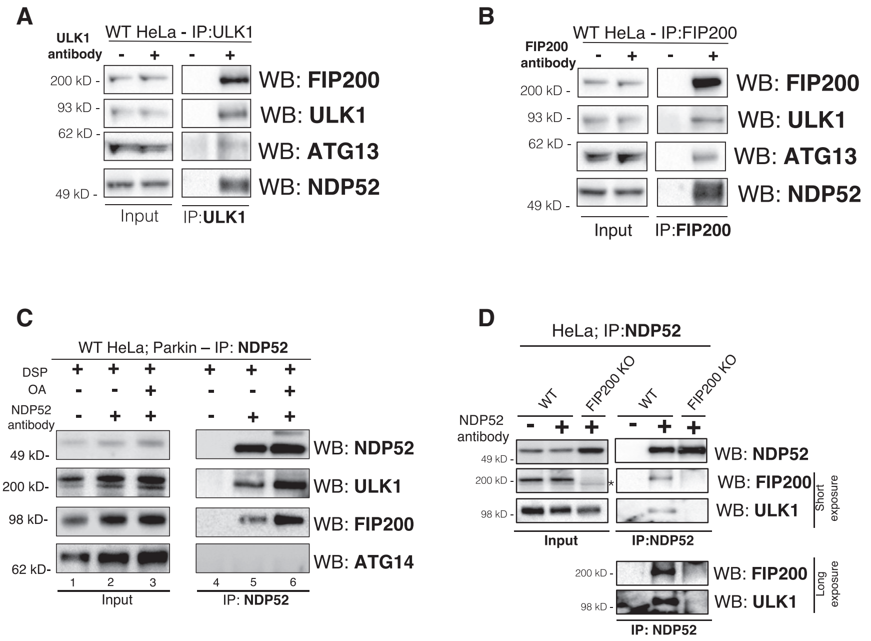

Figure 2. NDP52 Interacts with ULK1 Complex through FIP200

(A and B) Western analysis of immunoprecipitation assay of endogenous FIP200 (B) or ULK1 (A) in HeLa cells.

(C) Endogenous immunoprecipitation of NDP52 from WT HeLa cells treated with oligomycin/antimycin (OA) and Bafilomycin (200 nM; to prevent receptor degradation) for 3 h. Cells were crosslinked *in situ* using DSP prior to immunoprecipitation and western blot analysis.

(D) Western analysis of immunoprecipitation assay for NDP52 in either WT or FIP200 KO HeLa cells.

See also Figure S1.

Parkin-mediated mitophagy (Figures 2C and S1A). At endogenous NDP52 levels, the increase in the interaction of NDP52 and the ULK1 complex during mitophagy requires DSP crosslinking for clear detection. ATG14, a component of the VPS34 complex, does not appear to interact with NDP52 even under crosslinking conditions, suggesting some specificity of the association of NDP52 to the ULK1 complex (Figure 2C). In FIP200 KO cells, NDP52 no longer associates with ULK1, suggesting that NDP52 binds the complex through FIP200 (Figure 2D). This binding deficiency occurs even though FIP200 KO cells have much higher NDP52 protein levels (Figures 2D and S1B). These interactions were confirmed by complementary immunoprecipitation of ULK1 in WT or FIP200 KO cells (Figure S1C). Co-overexpression of FIP200 increased the association of exogenous NDP52 with ULK1 (Figure S1D).

Functional Analysis of NDP52-FIP200 Interaction

To corroborate the interaction of NDP52 and FIP200, we performed mutational mapping (Figure 3A). NDP52 contains 7 distinct domains (Xie et al., 2015; Verlhac et al., 2015). The deletion of the SKICH domain of NDP52, which is responsible for interactions with Nap1 and Sintbad (Thurston et al., 2009), inhibited the NDP52-FIP200 association (Figure 3B). In contrast, mutations in the LIR domains of NDP52 did not attenuate interaction with FIP200 (CLIR/LIR-like null; Figures 3B and 3C). Using the FIP200-binding-deficient NDP52 mutant, we tested the importance of the SKICH domain for NDP52-CID-mediated mitophagy. FIP200 was not recruited to the mitochondria when the CID assay was performed using FKBP-ΔSKICH-NDP52 (Figure S1E). Furthermore, we did not detect mitophagy after localizing a FKBP-ΔSKICH-NDP52 mutant to mitochondria, suggesting that the interaction of NDP52 with FIP200 is critical for the ability of NDP52 to initiate mitophagy (Figure 4A). Thus, the binding of NDP52 to FIP200 through the SKICH domain appears to be important for mitophagy induced by NDP52 cargo localization.

FIP200 is important for the integrity of the ULK1 complex by acting as a scaffold for its components (Hurley and Young, 2017). Comparing FIP200 truncation mutants revealed that only the C-terminal half of FIP200 has affinity for NDP52 (FIP200 C1: 800–1,591 aa; Figure 3D). A peptide containing the C-terminal leucine zipper domain of FIP200 is sufficient to co-precipitate NDP52 (FIP200 C2: 1,300–1,591 aa), whereas a C-terminal fragment excluding the leucine zipper domain is defective in binding NDP52 (FIP200 C3: 1,400–1,591 aa; Figure 3E). Further truncation of FIP200 revealed that a 127-aa peptide spanning the leucine zipper domain (LZ; 1,286–1,413 aa) is sufficient to interact with NDP52. Mutating the 4 leucines to alanines (LZA; L1371A, L1378A, L1392A, and L1385A) in the leucine zipper peptide abolishes this association (Figure 3F). Importantly, mutating the same 4 leucines to alanines within the leucine zipper of the full-length FIP200 also attenuates its binding to NDP52 (Figure 3G). A previous report showed that four contiguous point mutations in FIP200 at positions 582–585 from LFQL to AAAA selectively eliminated the starvation-induced autophagic function of FIP200 by preventing the interaction between FIP200 and ATG13 (Chen et al., 2016). We found that these mutations have no effect on the association of FIP200 and NDP52, suggesting that the interaction of FIP200 and NDP52 may be dissociable from the association of FIP200 with the ULK1 complex (Figure 3G). To validate the involvement of the leucine zipper domain of FIP200 in interacting with NDP52 in live cells, we expressed the WT leucine zipper (LZ: 1,286–1,413 aa) or the alanine mutant leucine zipper (LZA: 1,286–1,413 aa; L1371A, L1378A, L1392A, and L1385A) in cells stably expressing Parkin and NDP52. The leucine zipper peptide translocated with NDP52 to Parkin foci after Oligomycin and Antimycin (OA) treatment, whereas the 4-alanine mutant peptide did not (Figure 3H). Thus, FIP200 interacts with NDP52 through its leucine zipper domain.

NDP52 Mediates *De Novo* LC3-Independent Selective Autophagy

Ablation of the SKICH domain hinders NDP52-CID-induced mitophagy (Figure 4A). ΔSKICH NDP52 still interacts with

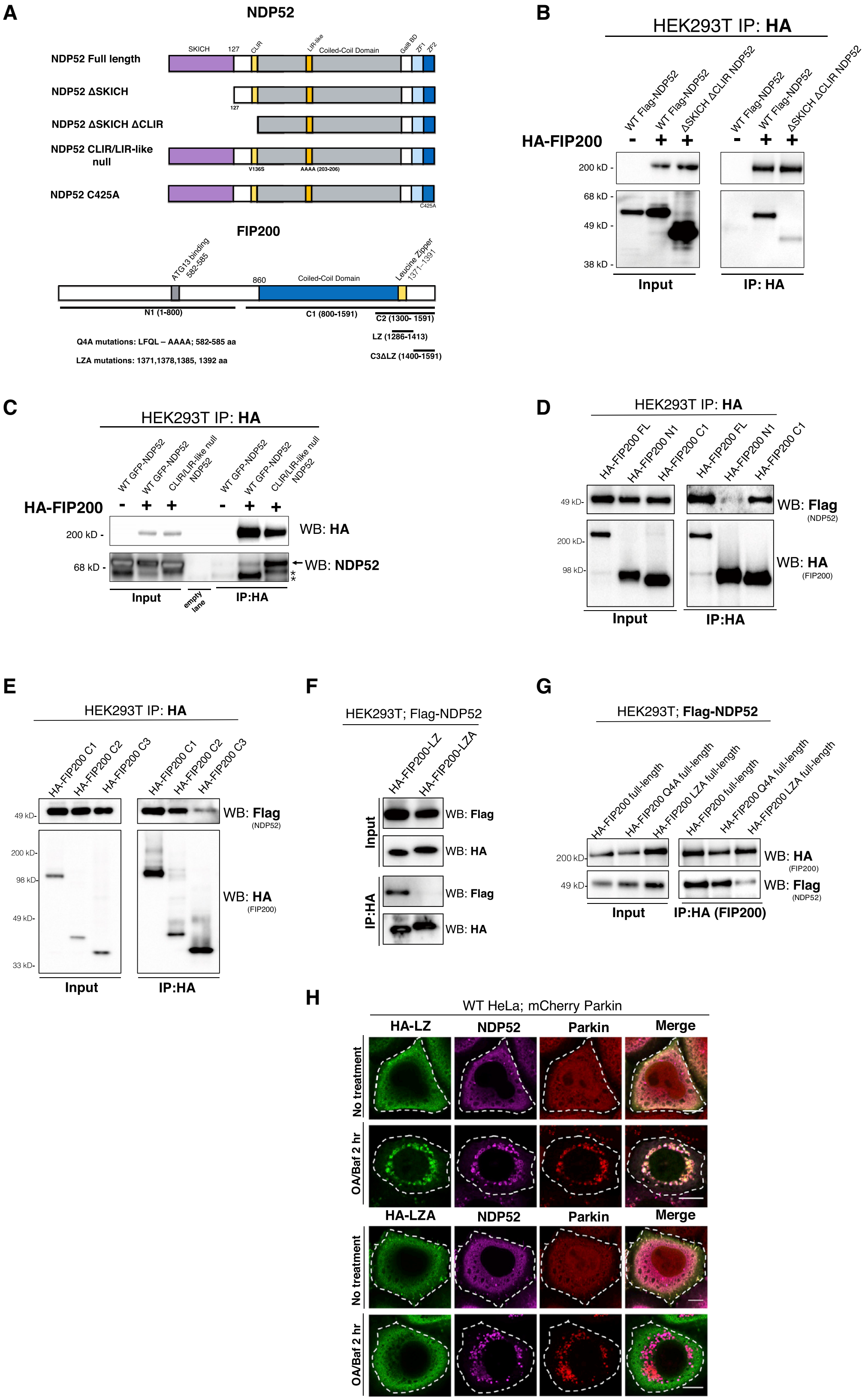

(legend on next page)

LC3/GABARAP proteins (von Muhlinen et al., 2012; Verlhac et al., 2015), suggesting that the interaction of NDP52 with LC3/GABARAPs may not be necessary for NDP52-mediated mitophagosome biogenesis. NDP52 binds ubiquitin chains through the ZF2 domain (Thurston et. al., 2009), and only the ZF2 domain is absolutely required for the recruitment of NDP52 to mitochondria after OA treatment, while SKICH or LC3-binding domains are not (Figure S2D). Consistently, only the ablation of the ZF2 domain of NDP52, but not SKICH or CLIR, blocked the ubiquitin binding of NDP52 (Figure S2E). These results show that the ubiquitin binding of NDP52 through ZF2 dictates its recruitment to cargo during Parkin-induced mitophagy, while the SKICH domain of NDP52 allows it to associate with the ULK1 complex (Figure 3B).

To directly test the importance of the LC3 binding of NDP52, we directed CLIR/LIR-like mutant NDP52 to the mitochondria using CID. This mutant induces mitophagy as efficiently as WT NDP52 (Figure 4B). Furthermore, upstream ATG proteins such as FIP200 and ULK1, as well as PI3P-binding protein DFCP1, are all able to localize to Parkin foci after inducing mitophagy with OA even in cells lacking all LC3/GABARAP proteins (Figure 4D; quantified in Figures 4E–4G). However, no mitolysosomes were observed after recruiting NDP52 or ULK1 to mitochondria of LC3/GABARAP KO cells, suggesting an inhibition of autophagosome-lysosome fusion events in the absence of LC3 proteins (Figures 4H and 4I). This interpretation is corroborated by the accumulation of DFCP1 foci in LC3/GABARAP KO cells at basal states (Figure 4C). Importantly, the block in Parkin-dependent mitophagy in OPTN, NDP52, and Tax1bp1 TKO cells can be rescued by complementation of either WT or LC3-binding-deficient NDP52 (Figure 4J). These data are consistent with previous findings showing that LC3/GABARAP proteins are not necessary for autophagosome targeting but rather in later autophagosome-lysosome fusion events (Nguyen et al., 2016). Furthermore, these results suggest that the capacity of NDP52 to bind LC3 proteins is not required for its function in localizing the ULK1 complex to mediate selective degradation of ubiquitinated cargo.

Synthetic FIP200-Binding Peptide Induces Selective Autophagy

If the localization of the ULK1 complex to specific cargo by NDP52 can initiate *de novo* autophagosome biogenesis, then other proteins capable of binding FIP200 may also induce mitophagy when artificially targeted to the mitochondria. ATG16L1 interacts with FIP200 (Dooley et al., 2014; Gammoh et al., 2013; Nishimura et al., 2013) and is part of the Atg12-Atg5 ubiquitin-like conjugation system important for the recruitment of LC3 to the phagophore. We generated a truncated ATG16L1 peptide (100–250 aa) containing only the FIP200-binding region fused to FKBP (Figure S3A), thus excluding the LC3-dependent roles of ATG16L1 from our analyses. Localizing the ATG16L1 peptide to the mitochondria induced mitophagy (Figure S3B). Indeed, introducing E230R/E241R mutations into the ATG16L1 peptide, which abrogated the interaction with FIP200 (Figure S3C; Dooley et al., 2014), prevented the induction of mitophagy (Figure S3B). These experiments using a synthetic FIP200-binding peptide confirm that localizing the ULK1 complex to cargo initiates selective autophagy.

Ectopic Localization of ULK1 on Cargo Drives Induction of Selective Autophagy Independent of Energy-Sensing Pathways

It was previously reported that ULK1 is activated by AMPK during mitophagy (Egan et al., 2011). Monitoring mitophagy in AMPK alpha 1/2 double-KO cells (AMPK DKO) after OA treatment revealed that these cells had no defect in PINK1/Parkin-mediated mitophagy by western blot or mito-mKeima FACS analysis (Figures 5A and 5B). Furthermore, 4 or 18 h of starvation did not induce mitophagy in either WT or AMPK DKO cells (Figures 5A and 5C). On the other hand, pharmacologic inhibition of ULK1/2 kinase fully inhibited OA-induced mitophagy, confirming that ULK1/2 kinase activity is required for PINK1/Parkin-mediated mitophagy (Figure 5D). Interestingly, unlike FIP200 KO cells, which displayed defective LC3 lipidation as well as aberrant p62 accumulation, the AMPK DKO U2OS cells had LC3 lipidation defects yet did not accumulate p62 basally (Figures S4A–S4D).

Figure 3. Mutational Dissection of NDP52-FIP200 Interaction

(A) Diagram of NDP52 mutation and deletions. ΔSKICH is a deletion from position 1–127. ΔSKICH ΔCLIR is a deletion from 1–148. CLIR null is a V136S mutation in the LC3C binding domain. LIR-like null pertains to AAAA substitution of DYWE at position 203–206 within the non-canonical LIR domain. C425A is a mutation in ubiquitin-binding ZF2 motif. Diagram of FIP200 mutations and deletions. Q4A refers to 4 alanine substitutions within the ATG13-binding domain. LZA refers to 4 alanine mutations in the leucine zipper domain.

(B) Western analysis of Flag-NDP52 WT and mutants (see Figure 3H for NDP52 mutation map), and HA-FIP200 were transiently transfected in HEK293T cells followed by HA immunoprecipitation.

(C) Western analysis of HA-FIP200 immunoprecipitation performed on HEK293T cells overexpressing HA-FIP200 and GFP-NDP52 or GFP-NDP52 CLIR/LIR-like null. Arrow indicates NDP52 band. Asterisks indicate background bands.

(D) Western blot analysis of HEK293T cells transfected with full-length, N1 (1–800), and C1 (800–1,591) HA-FIP200 co-transfected with Flag-NDP52, then subjected to HA immunoprecipitation.

(E) Western blot analysis after HA immunoprecipitation performed in HEK293T cells transfected with HA-FIP200 C1 (800–1,591), C2 (1,300–1,591), C3 (1,400–1,591) and Flag-NDP52.

(F) Western blot analysis after HA immunoprecipitation performed on HEK293T cells transfected with HA-LZ (1,286–1,413 of FIP200), HA-LZA (1,286–1,413 of FIP200; AAAA substitution at positions 1,371, 1,378, 1,385, and 1,392) and Flag-NDP52.

(G) Western blot analysis after HA immunoprecipitation performed on HEK293T cells transfected with full-length HA-FIP200, HA-FIP200 Q4A (full-length FIP200 with AAAA substitutions at positions 582–585), HA-FIP200 LZA (full-length FIP200 with AAAA substitution at positions 1,371, 1,378, 1,385, 1,392), and Flag-NDP52.

(H) Confocal imaging of WT HeLa cells stably expressing mCherry-Parkin and GFP-NDP52 and transiently transfected with WT FIP200 leucine zipper (HA-LZ) or alanine mutant (HA-LZA), then treated with OA for 3 h. Cells were then fixed and stained for HA. Scale bars: 10 μm.

See also Figures S1 and S2.

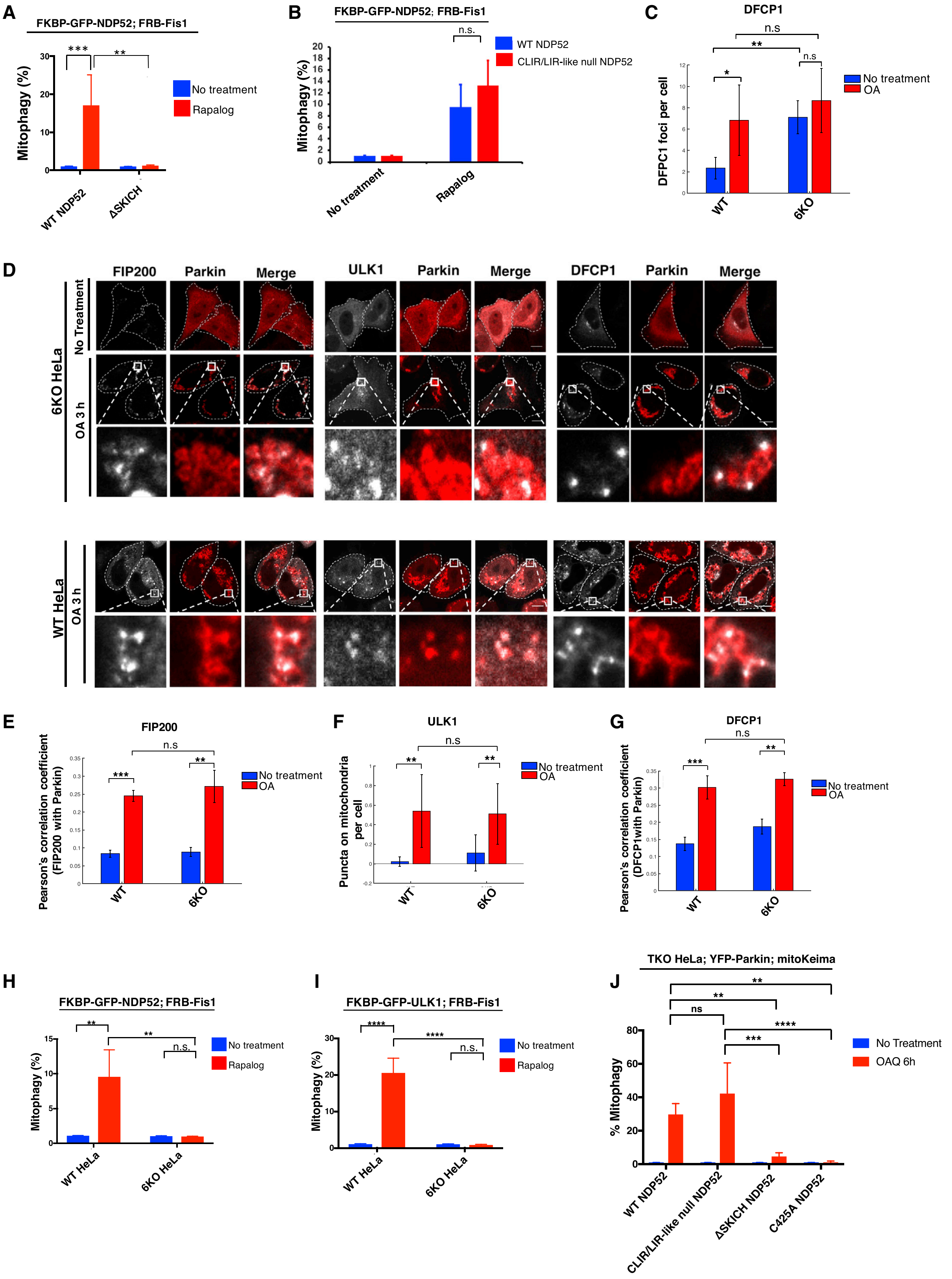

(legend on next page)

Starvation or Torin1 treatment of these AMPK DKO cells resulted in the degradation of p62, which was recovered by co-treatment with Bafilomycin. This observation is in contrast to loss of AMPK in mouse, where basal accumulation of p62 is observed (Egan et al., 2011).

The lack of involvement of AMPK in PINK1/Parkin-mediated mitophagy suggests that ULK1 may autoactivate directly on cargo. In yeast, this mode of activation has been suggested for Atg1 during cytosol-to-vacuole targeting (Cvt) and pexophagy (Kamber et al., 2015; Torggler et al., 2016). Targeting ULK1 kinase onto mitochondria by CID elicited mitophagy in both WT and AMPK DKO cells (Figure 5E). Introduction of a T180A mutation within the kinase loop of ULK1 completely prevented mitophagy following localization of ULK1 to the cargo (Figure 5F). This conserved threonine residue is an autophosphorylation site and is critical for ULK1 kinase activation (Bach et al., 2011). Other ULK1 autophosphorylation sites previously identified in a screen (Egan et al., 2015) did not affect ULK1-CID mitophagy (Figure S4E). Treatment of cells with an ULK1/2 inhibitor completely attenuated the ULK1-CID mitophagy (Figure 5G), suggesting that, although AMPK is not required, the activation of ULK1 is necessary for cargo degradation. The absence of AMPK also did not affect mitolysosome formation induced by the tethering of NDP52 to cargo (Figure 5H). Similarly, the FIP200-binding peptide derived from ATG16L1, when localized to mitochondria of WT or AMPK DKO cells, resulted in similar levels of mitophagy (Figures 5I and S3E). These results reveal that ULK1 localization to cargo organelles is sufficient to initiate selective autophagy even in the absence of AMPK.

mTOR phosphorylates ULK1 at S757 to inhibit its autophagic function when there is an abundance of intracellular energy (Kim et al., 2011). We thus tested how mTOR phosphorylation of ULK1 affects its ability to initiate autophagy when tethered to cargo. Inhibition of mTOR by Torin1 does not result in mitophagy (Figure 5J), consistent with the starvation results (Figure 5C). Overexpression of mTOR increases phosphorylation of ULK1 at S757 and another established mTOR substrate, p70S6K at T389. Both phosphorylation events are eliminated in the presence of Torin1 (Figure 5K). Tethering of the FIP200-binding peptide on the mitochondria triggers mitophagy despite the sustained phosphorylation of ULK1 at S757 due to mTOR overexpression as observed by western blot (Figure 5K). Using the more sensitive mito-mKeima assay, we observed that mTOR overexpression, rather than inhibiting, slightly increased the mitolysosome formation triggered by the tethering of FIP200-binding peptide or ULK1 itself on the mitochondria (Figures 5L and 5M). These results suggest that targeting of ULK1 on cargo, and the subsequent induction of selective autophagy, can circumvent mTOR inhibition during nutrient-replete states.

TBK1 Positively Regulates Mitophagy by Facilitating NDP52-ULK1 Complex Association

TBK1 phosphorylates selective autophagy receptors to increase ubiquitin binding and retention on cargo (Wild et al., 2011; Heo et al., 2015; Richter et al., 2016). Knocking out TBK1 in HeLa or HCT116 cells decreases the mitochondrial clearance rate following up to 24 h of OA treatment (Figures S5A–S5C) without altering the expression of ULK1 complex components (Figure S5D). However, after 3 h of OA treatment, the mitochondrial recruitment of NDP52 in TBK1 KO cells appears to be comparable to that of WT cells (Figures S2A–S2C), suggesting an additional role for TBK1.

The mitophagy induced by tethering NDP52 onto mitochondria is attenuated in TBK1 KO cells but not in autophagy receptor KO cells, further indicating that TBK1 has functions apart from stabilizing receptors on cargo (Figure 6A). This result is consistent with previous work in xenophagy showing that TBK1 is required for the recruitment of WIPI2 (Thurston et al., 2016). On the other hand, mitochondrial localization of ULK1 fully triggered mitophagy in the absence of TBK1 or autophagy receptors, indicating that direct cargo localization of ULK1 bypasses the function of NDP52 and TBK1 (Figure 6B). The association of NDP52 and FIP200 is decreased in TBK1 KO cells, revealing that TBK1 fosters the association of NDP52 with the ULK1 complex (Figure 6C), which could partially explain why targeting of NDP52 to mitochondria in TBK1 KO cells does not elicit mitophagy. Furthermore, pharmacological inhibition of TBK1 did not block the mitophagy induced by targeting FIP200-binding peptide to the mitochondria (Figure S3D), suggesting that TBK1 activity is specifically required for NDP52-CID mitophagy. NDP52- and ULK1-CID induced mitophagy is blocked in FIP200 KO cells, indicating that FIP200 is necessary for ULK1 activation on cargo (Figures 6A and 6B). Overexpression of WT NDP52 increased the rate of Parkin-mediated mitophagy after 3 or 6 h of OA treatment (Figures 6D and 6E). Mitophagy levels even after overexpression of NDP52 are lower in TBK1 KO cells (Figure 6D). The defect in mitophagy of TBK1 KO cells at 6 h of OA treatment is comparable to the defect in mitophagy in autophagy receptor KO cells. However, by 24 h of OA treatment, some mitophagy is detectable in TBK1 KO

Figure 4. NDP52 Mediates Autophagosome Biogenesis on Cargo Organelle Independent of LC3

(A and B) Quantification of FACS ratiometric analysis of mito-mKeima after 24 h treatment of Rapalog. HeLa cells stably expressing FRB-Fis1, mito-mKeima, and FKBP-GFP-NDP52 WT and ΔSKICH in (A) or FKBP-GFP-NDP52 CLIR/LIR-like null in (B).

(C) Quantification of DFCP1 foci. WT or 6KO (LC3/GABARAP hexa KO) cells expressing GFP-DFCP1 were analyzed at basal conditions or after 3 h OA.

(D) Confocal imaging of WT or 6KO HeLa cells transiently expressing GFP-ULK1, GFP-FIP200, or GFP-DFCP1, as well as HA-Parkin. Cells were immunostained for HA.

(E–G) Pearson's correlation or automated counting of overlapping puncta between FIP200 (E), ULK1 (F), or DFCP1 (G) on Parkin before or after OA treatment.

(H and I) Quantification of FACS ratiometric analysis of mito-mKeima. FKBP-GFP-ULK1 (I) or NDP52 (H) were stably expressed with FRB-Fis1 and mito-mKeima in WT or 6KO cells. Cells were then treated with Rapalog for 24 h.

(J) NDP52 WT and mutants, as well as mito-mKeima, were stably expressed in OPTN, NDP52, Tax1bp1 triple KO cells (TKO). Cells were treated with OA for 6 h then analyzed by FACS for mitophagy.

All FACS quantifications: n = 3. Data are represented as mean ± SD. p value: * = < 0.05; ** < 0.01; *** < 0.001; **** < 0.00001; ns., not significant. Scale bars: 10 μm.

See also Figures S2 and S5.

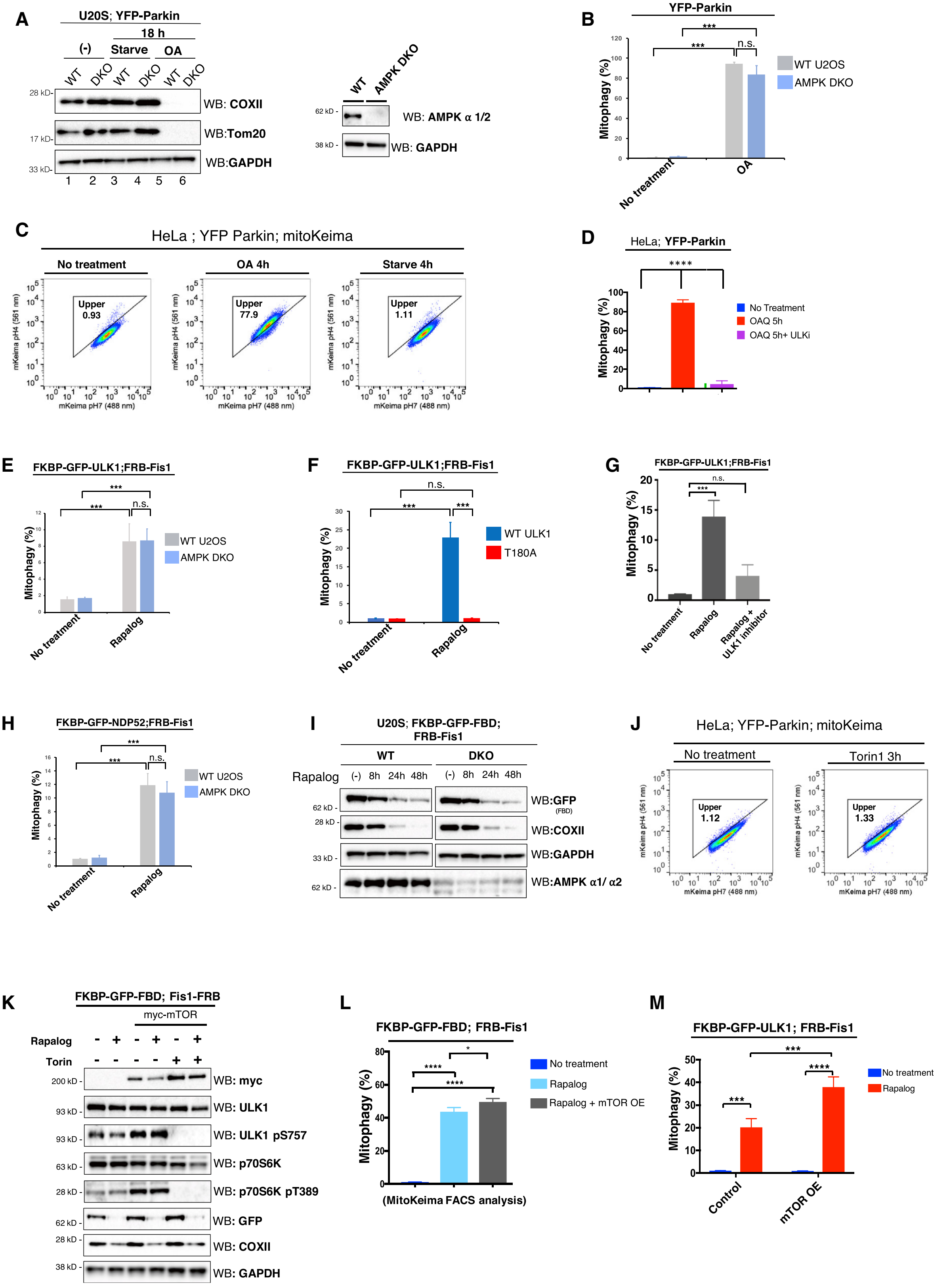

(legend on next page)

cells (Figure 6F), suggesting that TBK1 governs the rate of mitophagy but is not absolutely required for the process. TBK1 targeted to mitochondria induces mitophagy, whereas kinase-dead TBK1 does not (Figure 6G). Similarly, treatment with a TBK1 inhibitor prevents the induction of mitophagy after mitochondrial tethering of TBK1 (Figure 6H). Indeed, upon ectopic localization of TBK1 on mitochondria, the S172 autophosphorylation site of TBK1 can be detected on the mitochondria by immunofluorescence (Figure S6A; box a). In Rapalog-treated cells where TBK1 failed to translocate, no phosphorylated S172 is detected (Figure S6A, box b). In contrast, S172 phosphorylation is not observed after tethering of kinase-dead TBK1 on mitochondria (Figure S6B). Localizing TBK1 to mitochondria caused phosphorylation at S172 on the organelle, dependent on TBK1 kinase activity (Figures S6C and S6D). Consistently, western blot analysis reveals an increase in S172 phosphorylation after localizing TBK1 to mitochondria (Figure S6E). These results are consistent with biochemical and structural studies demonstrating TBK1 trans-autoactivation upon focal localization (Ma et al., 2012; Helgason et al., 2013).

The ability of TBK1-CID to initiate mitophagy is attenuated in the absence of mitophagy receptors OPTN, NDP52, and Tax1bp1, suggesting that both the kinase activity and interaction with receptor proteins are required for TBK1 to initiate mitophagy (Figure 6I). Consistently, ectopic mitochondrial placement of TBK1-ΔC terminus, which is unable to bind receptor proteins (Thurston et al., 2016), cannot trigger mitophagy (Figure 6J). Further, mitochondrially localized TBK1 is unable to induce mitophagy in the presence of ULK1/2 inhibitor, suggesting that TBK1 cargo localization causes upstream activation of ULK1 (Figure 6K). These results suggest that TBK1 modulates the ability of NDP52 to recruit and activate ULK1 on cargo by promoting NDP52-FIP200/ULK1 complex association (Figure 6L).

TBK1 Function Requires NDP52 during OA-Induced PINK1/Parkin Mitophagy

To further test the functional relationship between NDP52 and TBK1 during PINK1/Parkin-mediated mitophagy induced by mitochondrial depolarization, we engineered various chimeric TBK1/NDP52 proteins and tested which combinations will rescue the mitophagy defect in either TBK1 KO or autophagy receptor TKO cells (Figure S5E). WT TBK1, but not TBK1-ΔC terminus, rescues the mitophagy defect in TBK1 KO cells. However, when TBK1-ΔC terminus is fused to full-length NDP52, this chimera is now able to rescue mitophagy in TBK1 KO cells (Figure S5F). In autophagy receptor TKO cells, TBK1-ΔC-NDP52 rescues the mitophagy defect, whereas the TBK1-ΔC-ΔSKICH-NDP52 does not (Figure S5G), supporting the role of SKICH domain of NDP52 in associating with both TBK1 (Thurston et al., 2009) and FIP200 (Figure 3B). The ability of the TBK1-ΔC-ΔSKICH-NDP52 to rescue mitophagy in TBK1 KO cells is likely due to the presence of autophagy receptors that can compensate for the nonfunctional ΔSKICH-NDP52 (Figure S5F).

Peroxisome Localization of NDP52 or ULK1 Induces Pexophagy

We sought to determine if the ULK1 complex activation by mitochondrial targeting can be generalized to induce selective autophagic degradation of other organelles. Therefore, we localized ULK1, NDP52, and TBK1 to peroxisomes using CID by expressing FRB-PMP34 and FKBP fused to the protein of interest. We used catalase immunostaining as a measure of pexophagy. Catalase is normally a matrix-localized peroxisomal protein and thus appears in punctate structures in cells with intact peroxisomes. However, in the absence of peroxisomes, catalase mislocalizes to the cytosol and can be used as a proxy for pexophagy. Ectopic localization of NDP52 to peroxisomes resulted in peroxisomal loss, which was partially attenuated in cells

Figure 5. ULK1 Autoactivation by Ectopic Localization Elicits Mitophagy

(A) WT or AMPK DKO U2OS cells stably expressing YFP-Parkin were either treated with OA or starved in HBSS for 18 h followed by western blot analysis of mitochondrial proteins to measure mitophagy.

(B) Cells in (A) also stably expressing mito-mKeima were treated with OA for 6 h, then analyzed by FACS for mito-mKeima 561/488 nm ratio.

(C) FACS analysis plots of HeLa cells expressing YFP-Parkin and mito-mKeima after 4 h treatment of OA or starvation showing no mitophagy detected after starvation.

(D) Cells in (C) were treated with OA or OA with ULK1/2 inhibitor for 5 h followed by FACS analysis.

(E) Quantification of FACS ratiometric analysis of mito-mKeima of WT or AMPK DKO U2OS cells stably expressing FKBP-GFP-ULK1, FRB-Fis1, and mito-mKeima treated with Rapalog for 24 h.

(F) Quantification of FACS ratiometric analysis of mito-mKeima of HeLa cells expressing FKBP-GFP-ULK1 (WT or T180A), FRB-Fis1, and mito-mKeima treated with Rapalog for 24 h.

(G) FACS analysis of mito-mKeima. HeLa cells expressing FKBP-GFP-ULK1, FRB-Fis1, and mito-mKeima were treated with Rapalog for 24 h in the presence or absence of ULK1/2 inhibitor (MRT68921, 1 μM).

(H) Quantification of FACS mito-mKeima analysis of WT or AMPK DKO U2OS cells expressing FKBP-GFP-NDP52, FRB-Fis1, and mito-mKeima treated with Rapalog for 24 h.

(I) WT and AMPK DKO U2OS cells expressing FKBP-GFP-FBD, FRB-Fis1, and mito-mKeima were treated with Rapalog for 8, 24, and 48 h. COXII levels were then analyzed by western blot to measure mitophagy.

(J) FACS plot of mito-mKeima mitophagy analysis. Cells expressing YFP-Parkin and mito-mKeima were treated with Torin1 for 3 h.

(K) Western analysis of HeLa cells expressing FKBP-GFP-FBD, FRB-Fis1, and mito-mKeima, as well as myc-mTOR. Cells were treated with Rapalog or concurrently with Torin1 (1 μM) for 24 h.

(L) FACS analysis for mito-mKeima of cells expressing FKBP-GFP-FBD, FRB-Fis1, mito-mKeima, and myc-mTOR.

(M) HeLa expressing FKBP-GFP-ULK1, FRB-Fis1, and myc-mTOR were analyzed for mito-mKeima signal by FACS.

All FACS quantifications: n = 3 biological replicates. Data are represented as mean ± SD. p value: * = < 0.05; ** < 0.01; *** < 0.001; **** < 0.00001; ns., not significant.

See also Figures S3, S4, and S7.

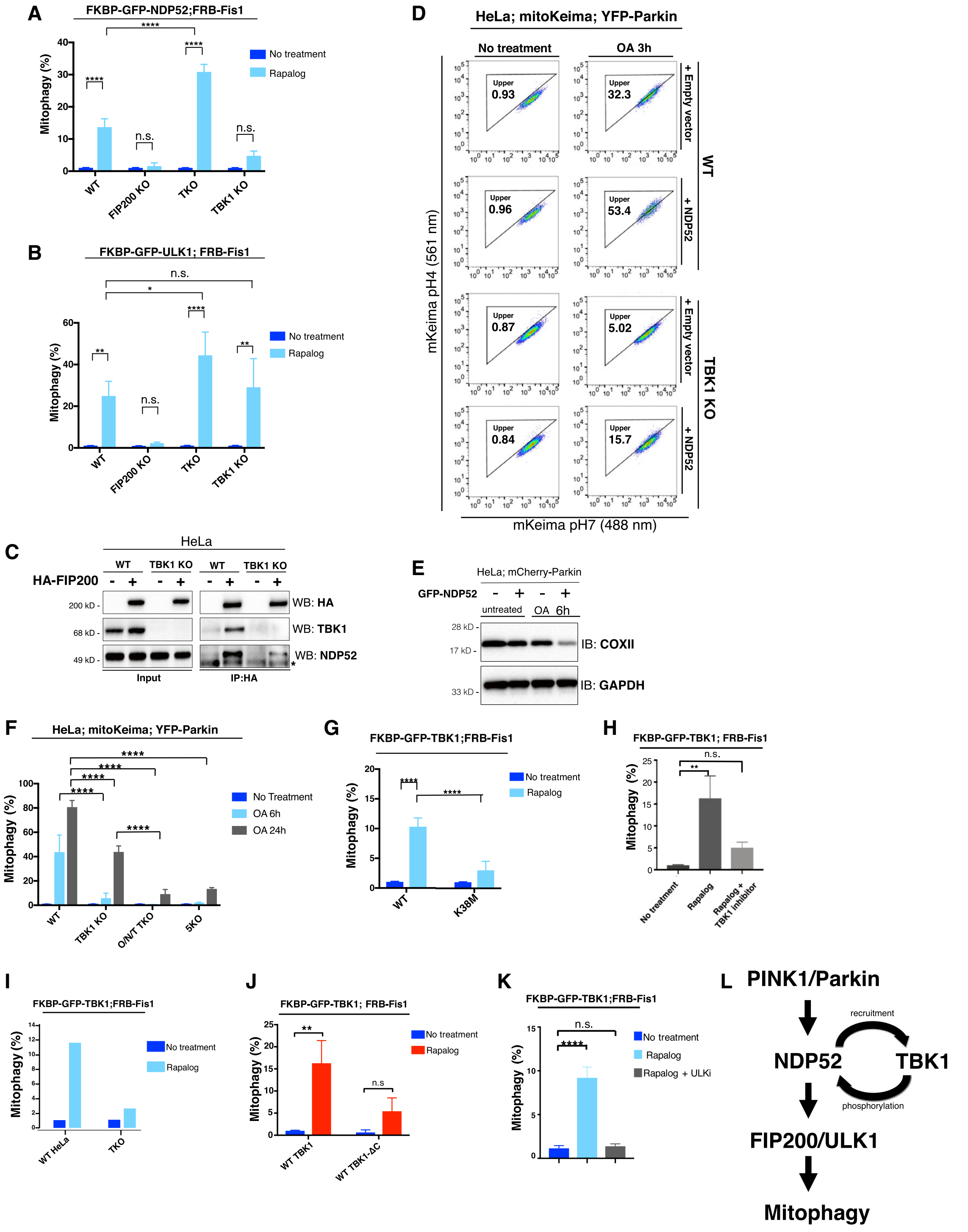

Figure 6. TBK1 Mediates NDP52-ULK1 Complex Mitophagy

(A) Quantification of FACS ratiometric analysis of mito-mKeima. WT, FIP200 KO, TKO (OPTN, NDP52 Tax1bp1 triple KO), or TBK1 KO cells stably expressing FKBP-GFP-NDP52, FRB-Fis1, and mito-mKeima treated with Rapalog for 24 h.

(B) Quantification of FACS analysis of mito-mKeima. WT and KO cells as in (A), stably expressing FKBP-GFP-ULK1, FRB-Fis1, and mito-mKeima treated with 24 h Rapalog.

(C) Western analysis of HA-FIP200 immunoprecipitation in WT or TBK1 KO HeLa cells. Endogenous NDP52 and TBK1 are immunoblotted.

(D) FACS plots of mito-mKeima ratiometric analysis. WT or TBK1 KO cells stably expressing mito-mKeima and YFP-Parkin, and transiently expressing BFP-NDP52 were treated with OA for 3 h.

(legend continued on next page)

lacking LC3/GABARAP proteins (Figures S7A and S7B). Peroxisomes also aggregated upon localization of NDP52 to the organelle in LC3/GABARAP KO cells (Figures S7A and S7B). Localizing ULK1 on peroxisomes caused their elimination, and this pexophagy was partially attenuated in LC3/GABARAP KO cells (Figure S7C). However, similar to the mitophagy results, ULK1 was able to bypass the requirement of the five autophagy receptors when directly targeted to peroxisomes (Figure S7C). On the other hand, consistent with our mitophagy findings, tethering TBK1 to peroxisomes of cells lacking autophagy receptor proteins did not induce pexophagy (Figures S7D and S7E). It is important to note that overexpression of peroxisome-localized proteins could result in interference of peroxisome biogenesis, which could explain the cytosolic catalase staining partially observed even in LC3/GABARAP KO cells (Figures S7A–S7C). Nonetheless, cytosolic catalase staining in WT cells is significantly higher than in LC3/GABARAP KO cells, indicating that pexophagy is indeed occurring after localizing NDP52 or ULK1 to peroxisomes. Expectedly, pexophagy by NDP52-CID is blocked in FIP200 KO cells (Figure S7F). Overall, these results corroborate the mitophagy results and suggest that ULK1 cargo localization and activation can drive the process of targeted autophagy for various organelles.

Chimeric Parkin Able to Bind the ULK1 Complex Functionally Compensates for Loss of TBK1 or Receptor Proteins during Mitophagy

Mitochondrial insult results in the activation of PINK1/Parkin, leading to the ubiquitination of mitochondrial outer membrane proteins. However, the chemically induced dimerization assays we utilized do not activate the PINK1/Parkin pathway and thus bypass cargo ubiquitination. To further test the model that TBK1 and NDP52 function downstream of ubiquitination pathways to recruit the ULK1 complex to cargo, we engineered a chimeric Parkin fused with an N-terminal FIP200-binding peptide (FBD-PARKIN), allowing this Parkin to associate to the ULK1 complex (Figure 7A). Cells lacking TBK1 or autophagy receptor proteins are defective in mitophagy after 6 h of OA treatment (Figure 6F). Stable expression of FBD-Parkin in either of these KO cells can rescue the mitophagy defect, bypassing the function of NDP52/TBK1 in recruiting the ULK1 complex during depolarization-induced mitophagy (Figure 7B). As a control, we fused an FBD mutant unable to bind FIP200 (E230R/E241R) to Parkin. This chimera initiates mitophagy in WT cells, due to the functional Parkin, but fails to rescue the mitophagy deficiency in either TBK1 or receptor KO cells (Figure 7C). Further, fusing FBD to a Parkin mutant defective in mitochondrial translocation (K211N; Koyano et al., 2013) also fails to rescue mitophagy in WT and KO cells (Figure 7D). These data further demonstrate that a critical function of TBK1 and NDP52 is to target the ULK1 complex to ubiquitinated cargo. The incomplete rescue of FBD-Parkin observed particularly in receptor 5KO cells, however, suggests that receptor proteins may have other functions apart from recruiting the ULK1 complex.

DISCUSSION

The basis for cargo discrimination during selective autophagy often highlights the role of autophagy receptor proteins, such as NDP52, and their ability to target preformed phagophores to cargo by binding LC3 (Kirkin et al., 2009; Birgisdottir et al., 2013). However, this model does not address how a cell can concomitantly increase the formation of phagophores when demand for selective autophagy increases. In order to increase autophagy flux and generate more phagophores, ULK1 kinase activity is upregulated (Mizushima et al., 2011). However, ULK1 is constitutively repressed by energy sensors, such as mTOR, during energy-replete cellular states (Herzig and Shaw, 2018; Papinski and Kraft, 2016; Kim et al., 2011). How ULK1 activity is amplified to overcome repression under nutrient-rich conditions to adapt to selective autophagy demands is unknown. Seminal work in yeast revealed that Atg1, the yeast ortholog of ULK1, can be locally activated at the vacuole during Cvt, where the presence of both cargo and receptor protein Atg19 is required for Atg1 activity (Kamber et al., 2015; Torggler et al., 2016). In agreement with this model, we found that ectopically localizing ULK1 to mitochondria or peroxisomes is sufficient to activate it and induce organelle degradation. AMPK is not required for ULK1 activation when ULK1 is targeted to cargo organelles, in distinction from starvation-induced macroautophagy. In addition, ULK1 autophosphorylation occurs independently of AMPK (Bach et al., 2011). We also found that mTOR phosphorylation of ULK1 had a minimal effect on cargo degradation when the ULK1 complex is tethered to cargo. A possible mechanism for ULK1 autoactivation is by homo-oligomerization, as reported for other kinases (Endicott et al., 2012; Helgason

(E) Western analysis. Cells stably expressing mCherry-Parkin with or without GFP-NDP52 were treated with OA for 6 h.
(F) Quantification of FACS ratiometric analysis of mito-mKeima. WT, FIP200 KO, TKO, or TBK1 KO cells, all stably expressing mito-mKeima and YFP-Parkin, were treated with OA for 6 or 24 h.
(G) Quantification of FACS ratiometric analysis of mito-mKeima. Cells stably expressing FRB-Fis1 and either WT FKBP-GFP-TBK1 or kinase-dead FKBP-GFP-TBK1 K38M were treated with Rapalog for 24 h.
(H) mito-mKeima FACS analysis of cells stably expressing FRB-Fis1 and FKBP-TBK1 after mitochondrial localization of TBK1 with Rapalog with or without ULK1/2 inhibitor, MRT68921 at 1 μM.
(I) Quantification of FACS ratiometric analysis of mito-mKeima of WT or TKO stably expressing FRB-Fis1 and WT FKBP-GFP-TBK1.
(J) FACS analysis of cells expressing WT FKBP-GFP-TBK1 or FKBP-GFP-TBK1 ΔC terminus (Δ 685–729), with FRB-FIs1 and mito-mKeima after 24 h of Rapalog treatment.
(K) Quantification of FACS ratiometric analysis of HeLa cells expressing FKBP-GFP-TBK1, FRB-Fis1, and mito-mKeima after 24 h of Rapalog treatment.
(L) Model of NDP52/TBK1-mediated recruitment of ULK1 complex during mitophagy resulting from bypass experiments.
All FACS quantifications: n = 3 biological replicates, except in (F) n = 2. Data are represented as mean ± SD. p value: * = < 0.05; ** < 0.01; *** < 0.001; **** < 0.00001; ns., not significant.
See also Figures S5, S6, and S7.

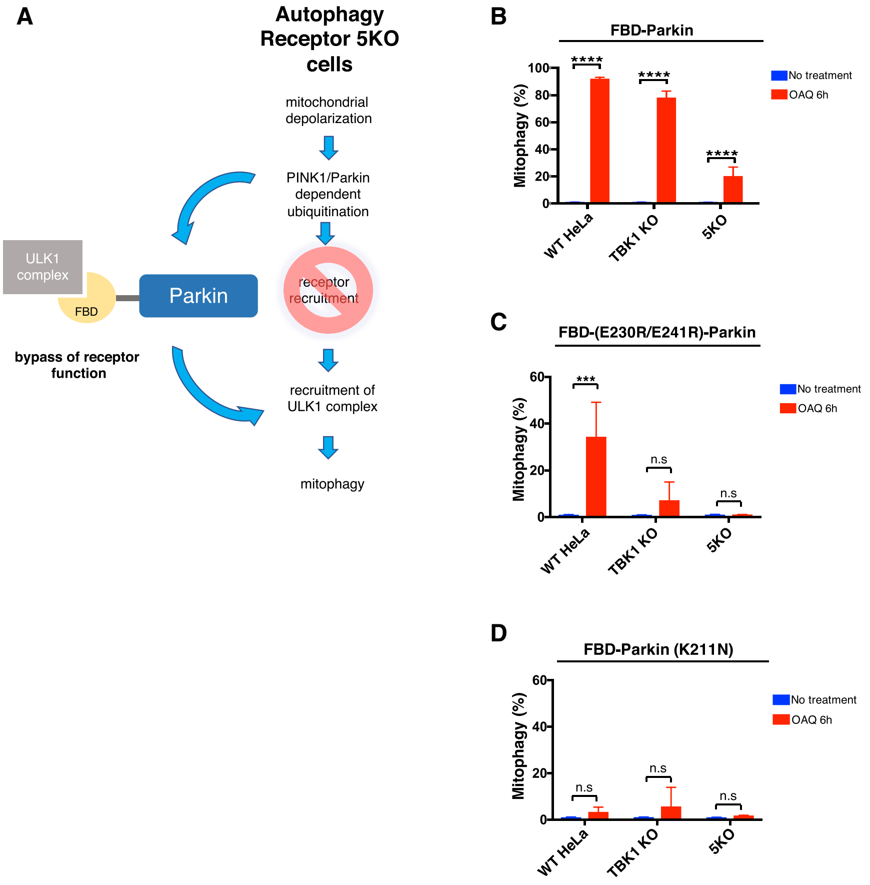

Figure 7. Chimeric FBD-Parkin Can Compensate for Loss of TBK1 or Receptor Proteins during Mitophagy

(A) Model for the bypass experiment performed using FIP200-binding peptide-Parkin (FBD-Parkin) in WT, TBK1 KO, or 5KO (OPTN, NDP52, Tax1bp1, NBR1, p62) cells.

(B) Mitophagy analysis by FACS. WT HeLa cells, TBK1 KO, or 5KO cells expressing mito-mKeima and FBD-Parkin.

(C and D) (C) FBD-Parkin (E230R/E241R) or (D) FBD-Parkin (K211N) were treated with OA and QVD for 6 h.

All FACS quantifications: n = 3 biological replicates. Data are represented as mean ± SD. p value: * = < 0.05; ** < 0.01; *** < 0.001; **** < 0.00001; ns., not significant.

See also Figure S3.

et al., 2013). Indeed, Atg1 activity is controlled by its local clustering (Torggler et al., 2016), consistent with our conclusion that increasing the local concentration of ULK1 on cargo is sufficient to drive autophagic induction, overcoming the repression of autophagy under nutrient-rich conditions.

Experimental tethering of NDP52 on mitochondria and peroxisomes promoted their elimination and depended on the association of NDP52 with the ULK1 complex via FIP200. Furthermore, rescue experiments in receptor knockout cell lines revealed that the LC3-binding capacity of NDP52 is dispensable for its function during mitophagy. Thus, it is likely that NDP52, rather than recruiting preformed phagophores to cargo, instead targets the ULK1 complex to initiate autophagosome biogenesis directly on the cargo. This interpretation is consistent with recent studies showing that cargo receptors are important for the recruitment of the ULK1 complex, as well as other downstream ATG machinery, during mitophagy (Lazarou et al., 2015) and that LC3/GABARAP proteins are dispensable for proper targeting of autophagosomes but are instead important for autophagosome fusion with lysosomes (Nguyen et al., 2016; Pontano Vaites et al., 2017). In neurons, LC3 foci appear in distal axons only after induction of Parkin-mediated mitophagy. Before triggering mitophagy, LC3 foci were not detected along axons, consistent with the model that autophagosomes are generated on demand during selective autophagy (Ashrafi et al., 2014). Nevertheless, it is evident that LC3/GABARAP proteins fulfill an essential role for proper maturation of autophagosomes, since mitophagosomes expanded more slowly and are smaller in diameter in LC3/GABARAP KO cells (Nguyen et al., 2016). LIR-binding domains are present or are predicted to be present among many ATG and ATG-related proteins (Alemu et al., 2012; Kraft et al., 2012; Kalvari et al., 2014). This might conceivably be how LC3 proteins mediate phagophore expansion during selective autophagy. Furthermore, it is highly likely that other receptors, such as OPTN, Tax1bp1, NBR1, and p62, may have activities overlapping with those of NDP52 in targeting upstream ATG components, apart from LC3, to various organelles during selective autophagy.

It is worth noting that the degree of mitophagy achieved by tethering single proteins on mitochondria is low in comparison to that caused by depolarizing agents. This is likely because the chemically induced dimerization experiments do not activate Parkin, while mitochondrial depolarizers do. Parkin ubiquitination of outer mitochondrial membrane (OMM) proteins is critical for the downstream recruitment of proteins that positively regulate mitophagy. For example, p97/VCP is recruited to mitochondria after Parkin activation to degrade Parkin-ubiquitinated OMM proteins (Tanaka et al., 2010). This OMM remodeling event is important for efficient mitophagy. Parkin recruits other factors, such as RABGEF1, that likely accelerate the mitophagosome membrane assembly (Yamano et al., 2018). Thus, other molecular events that depend on Parkin are missing in our experimental setup, possibly decreasing the total efficiency of mitophagy.

NDP52 concentrates the ULK1 complex on ubiquitinated cargo by binding FIP200. ATG16L1 also interacts with FIP200, suggesting that ATG16L1 may promote crosstalk between the later-stage LC3 lipidation pathway with the more-upstream ULK1 complex (Gammoh et al., 2013; Nishimura et al., 2013). We were able to experimentally validate our findings with NDP52 using a FIP200-binding peptide derived from ATG16L1 that mimics NDP52 recruitment and activation of ULK1 on cargo. Pharmacologic

inhibition of ULK1 completely attenuates mitophagy caused by ectopic localization of NDP52, the ATG16L1 peptide, or ULK1 itself to mitochondria, whereas loss of AMPK or overexpression of mTOR did not affect mitophagy induced by dimerization of these proteins to cargo. These findings corroborate the model that NDP52 induces ULK1 activation directly on the cargo.

TBK1 increases the affinity of receptor proteins to ubiquitin chains on cargo, leading to their increased retention on cargo (Heo et al., 2015; Richter et al., 2016). However, TBK1 may encompass a broader role in autophagosome biogenesis (Thurston et al., 2016; Pilli et al., 2012). Indeed, our experiments indicate that TBK1 functions in promoting the association of NDP52 with FIP200, possibly through the phosphorylation of NDP52, a known TBK1 substrate. However, inhibition of TBK1 did not affect the mitophagy induced by the FIP200-binding peptide, suggesting that TBK1 selectively mediates the interaction between NDP52 and FIP200. Interestingly, localizing TBK1 to mitochondria and peroxisomes induces the degradation of the respective organelles, which requires TBK1 kinase activity. Mitochondrial and peroxisomal localization of TBK1 did not trigger clearance of either organelle in cells lacking autophagy receptors, suggesting that TBK1 function during autophagy occurs via the autophagy receptors. Structural studies show that receptor-mediated focal localization sufficiently activates TBK1 through trans-autophosphorylation (Ma et al., 2012; Helgason et al., 2013). Thus, the activation mechanisms of TBK1 and ULK1 may share the common feature of proximity-induced activation. Here, we provide evidence that the localization of the ULK1 complex by NDP52 and TBK1 on cargo couples the activation and spatial regulation of ULK1 independently of AMPK and mTOR. This model simultaneously resolves the timing and positioning of selective autophagy induction.

STAR★METHODS

Detailed methods are provided in the online version of this paper and include the following:

- KEY RESOURCES TABLE
- CONTACT FOR REAGENT AND RESOURCE SHARING
- EXPERIMENTAL MODEL AND SUBJECT DETAILS
  - Cell Lines
- METHOD DETAILS
  - Knock out line generation using CRISPR/Cas9 gene editing
  - Cloning and stable cell line generation
  - Western Analyses
  - Immunoprecipitation, GFP-TRAP, HA and Flag beads precipitation
  - Immunofluorescence microscopy
  - Image Analysis
  - Mitochondrial recruitment of proteins during Parkin-mediated mitophagy
  - Chemically inducible dimerization assays
  - Mitophagy assay with mito-mKeima via fluorescence activated cytometer
  - Pexophagy assay
- QUANTIFICATION AND STATISTICAL ANALYSIS

SUPPLEMENTAL INFORMATION

Supplemental Information can be found online with this article at https://doi.org/10.1016/j.molcel.2019.02.010.

ACKNOWLEDGMENTS

We would like to thank the laboratory of Michael Lazarou (Monash Biomedicine Discovery Institute) for sharing the LC3/GABARAP KO cells and the laboratory of Reuben Shaw (Salk Institute) for the U2OS AMPK KO cells. We would also like to thank the other members of the Youle lab for providing inspiration and expertise through scientific conversations and Dr. Jeff Kowalak of the NIH/NINDS Clinical Proteomics Facility for instrument support. The authors would like to thank Hetal Shah for her help with the graphical abstract. This work is supported by the NINDS Intramural Research Program (R.J.Y.), MRC U105170648 (F.R), and the Wellcome Trust WT104752MA (F.R).

AUTHOR CONTRIBUTIONS

The project was conceptualized by J.N.S.V., C.W., F.R., and R.J.Y. Experiments and data analyses were performed by J.N.S.V., C.W., E.B., and L.H. Instrumentation and support on data analysis for FACS were contributed by D.M. Data interpretation was done by J.N.S.V., C.W., G.S., F.R., and R.J.Y. The manuscript was written by J.N.S.V. and R.J.Y and edited by C.W., G.S., and F.R. Supervision of the project was provided by G.S. and R.J.Y.

DECLARATION OF INTERESTS

The authors declare no competing interests.

Received: August 6, 2018
Revised: December 26, 2018
Accepted: February 6, 2019
Published: March 7, 2019

REFERENCES

Alemu, E.A., Lamark, T., Torgersen, K.M., Birgisdottir, A.B., Larsen, K.B., Jain, A., Olsvik, H., Øvervatn, A., Kirkin, V., and Johansen, T. (2012). ATG8 family proteins act as scaffolds for assembly of the ULK complex: sequence requirements for LC3-interacting region (LIR) motifs. J. Biol. Chem. *287*, 39275–39290.

Alers, S., Löffler, A.S., Wesselborg, S., and Stork, B. (2012). The incredible ULKs. Cell Commun. Signal. *10*, 7.

Ashrafi, G., Schlehe, J.S., LaVoie, M.J., and Schwarz, T.L. (2014). Mitophagy of damaged mitochondria occurs locally in distal neuronal axons and requires PINK1 and Parkin. J. Cell Biol. *206*, 655–670.

Bach, M., Larance, M., James, D.E., and Ramm, G. (2011). The serine/threonine kinase ULK1 is a target of multiple phosphorylation events. Biochem. J. *440*, 283–291.

Behrends, C., and Fulda, S. (2012). Receptor proteins in selective autophagy. Int. J. Cell Biol. *2012*, 673290.

Behrends, C., Sowa, M.E., Gygi, S.P., and Harper, J.W. (2010). Network organization of the human autophagy system. Nature *466*, 68–76.

Bento, C.F., Renna, M., Ghislat, G., Puri, C., Ashkenazi, A., Vicinanza, M., Menzies, F.M., and Rubinsztein, D.C. (2016). Mammalian autophagy: how does it work? Annu. Rev. Biochem. *85*, 685–713.

Birgisdottir, Å.B., Lamark, T., and Johansen, T. (2013). The LIR motif - crucial for selective autophagy. J. Cell Sci. *126*, 3237–3247.

Burman, J.L., Pickles, S., Wang, C., Sekine, S., Vargas, J.N.S., Zhang, Z., Youle, A.M., Nezich, C.L., Wu, X., Hammer, J.A., and Youle, R.J. (2017). Mitochondrial fission facilitates the selective mitophagy of protein aggregates. J. Cell Biol. *216*, 3231–3247.

Cantó, C., Gerhart-Hines, Z., Feige, J.N., Lagouge, M., Noriega, L., Milne, J.C., Elliott, P.J., Puigserver, P., and Auwerx, J. (2009). AMPK regulates energy

expenditure by modulating NAD+ metabolism and SIRT1 activity. Nature *458*, 1056–1060.

Chen, S., Wang, C., Yeo, S., Liang, C.-C., Okamoto, T., Sun, S., Wen, J., and Guan, J.-L. (2016). Distinct roles of autophagy-dependent and -independent functions of FIP200 revealed by generation and analysis of a mutant knock-in mouse model. Genes Dev. *30*, 856–869.

Dikic, I., and Elazar, Z. (2018). Mechanism and medical implications of mammalian autophagy. Nat. Rev. Mol. Cell Biol. *19*, 349–364.

Dooley, H.C., Razi, M., Polson, H.E.J., Girardin, S.E., Wilson, M.I., and Tooze, S.A. (2014). WIPI2 links LC3 conjugation with PI3P, autophagosome formation, and pathogen clearance by recruiting Atg12-5-16L1. Mol. Cell *55*, 238–252.

Egan, D.F., Shackelford, D.B., Mihaylova, M.M., Gelino, S., Kohnz, R.A., Mair, W., Vasquez, D.S., Joshi, A., Gwinn, D.M., Taylor, R., et al. (2011). Phosphorylation of ULK1 (hATG1) by AMP-activated protein kinase connects energy sensing to mitophagy. Science *331*, 456–461.

Egan, D.F., Chun, M.G.H., Vamos, M., Zou, H., Rong, J., Miller, C.J., Lou, H.J., Raveendra-Panickar, D., Yang, C.-C., Sheffler, D.J., et al. (2015). Small molecule inhibition of the autophagy kinase ULK1 and identification of ULK1 substrates. Mol. Cell *59*, 285–297.

Endicott, J.A., Noble, M.E.M., and Johnson, L.N. (2012). The structural basis for control of eukaryotic protein kinases. Annu. Rev. Biochem. *81*, 587–613.

Gammoh, N., Florey, O., Overholtzer, M., and Jiang, X. (2013). Interaction between FIP200 and ATG16L1 distinguishes ULK1 complex-dependent and -independent autophagy. Nat. Struct. Mol. Biol. *20*, 144–149.

Garcia, D., and Shaw, R.J. (2017). AMPK: mechanisms of cellular energy sensing and restoration of metabolic balance. Mol. Cell *66*, 789–800.

Gatica, D., Lahiri, V., and Klionsky, D.J. (2018). Cargo recognition and degradation by selective autophagy. Nat. Cell Biol. *20*, 233–242.

Helgason, E., Phung, Q.T., and Dueber, E.C. (2013). Recent insights into the complexity of Tank-binding kinase 1 signaling networks: the emerging role of cellular localization in the activation and substrate specificity of TBK1. FEBS Lett. *587*, 1230–1237.

Heo, J.-M., Ordureau, A., Paulo, J.A., Rinehart, J., and Harper, J.W. (2015). The PINK1-PARKIN mitochondrial ubiquitylation pathway drives a program of OPTN/NDP52 recruitment and TBK1 activation to promote mitophagy. Mol. Cell *60*, 7–20.

Herzig, S., and Shaw, R.J. (2018). AMPK: guardian of metabolism and mitochondrial homeostasis. Nat. Rev. Mol. Cell Biol. *19*, 121–135.

Hurley, J.H., and Young, L.N. (2017). Mechanisms of autophagy initiation. Annu. Rev. Biochem. *86*, 225–244.

Itakura, E., and Mizushima, N. (2010). Characterization of autophagosome formation site by a hierarchical analysis of mammalian Atg proteins. Autophagy *6*, 764–776.

Itakura, E., Kishi-Itakura, C., Koyama-Honda, I., and Mizushima, N. (2012). Structures containing Atg9A and the ULK1 complex independently target depolarized mitochondria at initial stages of Parkin-mediated mitophagy. J. Cell Sci. *125*, 1488–1499.

Jäger, S., Handschin, C., St-Pierre, J., and Spiegelman, B.M. (2007). AMP-activated protein kinase (AMPK) action in skeletal muscle via direct phosphorylation of PGC-1alpha. Proc. Natl. Acad. Sci. USA *104*, 12017–12022.

Kalvari, I., Tsompanis, S., Mulakkal, N.C., Osgood, R., Johansen, T., Nezis, I.P., and Promponas, V.J. (2014). iLIR: A web resource for prediction of Atg8-family interacting proteins. Autophagy *10*, 913–925.

Kamber, R.A., Shoemaker, C.J., and Denic, V. (2015). Receptor-bound targets of selective autophagy use a scaffold protein to activate the Atg1 kinase. Mol. Cell *59*, 372–381.

Kane, L.A., Lazarou, M., Fogel, A.I., Li, Y., Yamano, K., Sarraf, S.A., Banerjee, S., and Youle, R.J. (2014). PINK1 phosphorylates ubiquitin to activate Parkin E3 ubiquitin ligase activity. J Cell Biol *205*, 143–153.

Kim, J., Kundu, M., Viollet, B., and Guan, K.-L. (2011). AMPK and mTOR regulate autophagy through direct phosphorylation of Ulk1. Nat. Cell Biol. *13*, 132–141.

Kirkin, V., McEwan, D.G., Novak, I., and Dikic, I. (2009). A role for ubiquitin in selective autophagy. Mol. Cell *34*, 259–269.

Koyano, F., Okatsu, K., Ishigaki, S., Fujioka, Y., Kimura, M., Sobue, G., Tanaka, K., and Matsuda, N. (2013). The principal PINK1 and Parkin cellular events triggered in response to dissipation of mitochondrial membrane potential occur in primary neurons. Genes Cells *18*, 672–681.

Kraft, C., Kijanska, M., Kalie, E., Siergiejuk, E., Lee, S.S., Semplicio, G., Stoffel, I., Brezovich, A., Verma, M., Hansmann, I., et al. (2012). Binding of the Atg1/ULK1 kinase to the ubiquitin-like protein Atg8 regulates autophagy. EMBO J. *31*, 3691–3703.

Lazarou, M., Sliter, D.A., Kane, L.A., Sarraf, S.A., Wang, C., Burman, J.L., Sideris, D.P., Fogel, A.I., and Youle, R.J. (2015). The ubiquitin kinase PINK1 recruits autophagy receptors to induce mitophagy. Nature *524*, 309–314.

Ma, X., Helgason, E., Phung, Q.T., Quan, C.L., Iyer, R.S., Lee, M.W., Bowman, K.K., Starovasnik, M.A., and Dueber, E.C. (2012). Molecular basis of Tank-binding kinase 1 activation by transautophosphorylation. Proc. Natl. Acad. Sci. USA *109*, 9378–9383.

Mizushima, N., Yoshimori, T., and Ohsumi, Y. (2011). The role of Atg proteins in autophagosome formation. Annu. Rev. Cell Dev. Biol. *27*, 107–132.

Moore, A.S., and Holzbaur, E.L.F. (2016). Dynamic recruitment and activation of ALS-associated TBK1 with its target optineurin are required for efficient mitophagy. Proc. Natl. Acad. Sci. USA *113*, E3349–E3358.

Nguyen, T.N., Padman, B.S., Usher, J., Oorschot, V., Ramm, G., and Lazarou, M. (2016). Atg8 family LC3/GABARAP proteins are crucial for autophagosome-lysosome fusion but not autophagosome formation during PINK1/Parkin mitophagy and starvation. J. Cell Biol. *215*, 857–874.

Nishimura, T., Kaizuka, T., Cadwell, K., Sahani, M.H., Saitoh, T., Akira, S., Virgin, H.W., and Mizushima, N. (2013). FIP200 regulates targeting of Atg16L1 to the isolation membrane. EMBO Rep. *14*, 284–291.

Papinski, D., and Kraft, C. (2016). Regulation of autophagy by signaling through the Atg1/ULK1 complex. J. Mol. Biol. *428* (9 Pt A), 1725–1741.

Pilli, M., Arko-Mensah, J., Ponpuak, M., Roberts, E., Master, S., Mandell, M.A., Dupont, N., Ornatowski, W., Jiang, S., Bradfute, S.B., et al. (2012). TBK-1 promotes autophagy-mediated antimicrobial defense by controlling autophagosome maturation. Immunity *37*, 223–234.

Pontano Vaites, L., Paulo, J.A., Huttlin, E.L., and Harper, J.W. (2017). Systematic analysis of human cells lacking ATG8 proteins uncovers roles for GABARAPs and the CCZ1/MON1 regulator C18orf8/RMC1 in macro and selective autophagic flux. Mol. Cell. Biol. Published online October 16, 2017. MCB.00392–17.

Randow, F., and Youle, R.J. (2014). Self and nonself: how autophagy targets mitochondria and bacteria. Cell Host Microbe *15*, 403–411.

Richter, B., Sliter, D.A., Herhaus, L., Stolz, A., Wang, C., Beli, P., Zaffagnini, G., Wild, P., Martens, S., Wagner, S.A., et al. (2016). Phosphorylation of OPTN by TBK1 enhances its binding to Ub chains and promotes selective autophagy of damaged mitochondria. Proc. Natl. Acad. Sci. USA *113*, 4039–4044.

Russell, R.C., Yuan, H.-X., and Guan, K.-L. (2014). Autophagy regulation by nutrient signaling. Cell Res. *24*, 42–57.

Sun, N., Malide, D., Liu, J., Rovira, I.I., Combs, C.A., and Finkel, T. (2017). A fluorescence-based imaging method to measure in vitro and in vivo mitophagy using mt-Keima. Nat. Protoc. *12*, 1576–1587.

Tanaka, A., Cleland, M.M., Xu, S., Narendra, D.P., Suen, D.-F., Karbowski, M., and Youle, R.J. (2010). Proteasome and p97 mediate mitophagy and degradation of mitofusins induced by Parkin. J. Cell Biol. *191*, 1367–1380.

Thurston, T.L.M., Ryzhakov, G., Bloor, S., von Muhlinen, N., and Randow, F. (2009). The TBK1 adaptor and autophagy receptor NDP52 restricts the proliferation of ubiquitin-coated bacteria. Nat. Immunol. *10*, 1215–1221.

Thurston, T.L., Boyle, K.B., Allen, M., Ravenhill, B.J., Karpiyevich, M., Bloor, S., Kaul, A., Noad, J., Foeglein, A., Matthews, S.A., et al. (2016). Recruitment of TBK1 to cytosol-invading Salmonella induces WIPI2-dependent antibacterial autophagy. EMBO J. *35*, 1779–1792.

Tooze, S.A. (2013). Current views on the source of the autophagosome membrane. Essays Biochem. *55*, 29–38.

Torggler, R., Papinski, D., Brach, T., Bas, L., Schuschnig, M., Pfaffenwimmer, T., Rohringer, S., Matzhold, T., Schweida, D., Brezovich, A., and Kraft, C. (2016). Two independent pathways within selective autophagy converge to activate Atg1 kinase at the vacuole. Mol. Cell *64*, 221–235.

Toyama, E.Q., Herzig, S., Courchet, J., Lewis, T.L., Loson, O.C., Hellberg, K., Young, N.P., Chen, H., Polleux, F., Chan, D.C., et al. (2016). AMP-activated protein kinase mediates mitochondrial fission in response to energy stress. Science *351*, 275–281.

Verlhac, P., Grégoire, I.P., Azocar, O., Petkova, D.S., Baguet, J., Viret, C., and Faure, M. (2015). Autophagy receptor NDP52 regulates pathogen-containing autophagosome maturation. Cell Host Microbe *17*, 515–525.

von Muhlinen, N., Akutsu, M., Ravenhill, B.J., Foeglein, Á., Bloor, S., Rutherford, T.J., Freund, S.M.V., Komander, D., and Randow, F. (2012). LC3C, bound selectively by a noncanonical LIR motif in NDP52, is required for antibacterial autophagy. Mol. Cell *48*, 329–342.

Wang, J., Huo, K., Ma, L., Tang, L., Li, D., Huang, X., Yuan, Y., Li, C., Wang, W., Guan, W., et al. (2011). Toward an understanding of the protein interaction network of the human liver. Mol. Syst. Biol. *7*, 536.

Wesselborg, S., and Stork, B. (2015). Autophagy signal transduction by ATG proteins: from hierarchies to networks. Cell. Mol. Life Sci. *72*, 4721–4757.

Wild, P., Farhan, H., McEwan, D.G., Wagner, S., Rogov, V.V., Brady, N.R., Richter, B., Korac, J., Waidmann, O., Choudhary, C., et al. (2011). Phosphorylation of the autophagy receptor optineurin restricts salmonella growth. Science *333*, 228–233.

Xie, X., Li, F., Wang, Y., Wang, Y., Lin, Z., Cheng, X., Liu, J., Chen, C., and Pan, L. (2015). Molecular basis of ubiquitin recognition by the autophagy receptor CALCOCO2. Autophagy *11*, 1775–1789.

Yamano, K., Wang, C., Sarraf, S.A., Münch, C., Kikuchi, R., Noda, N.N., Hizukuri, Y., Kanemaki, M.T., Harper, W., Tanaka, K., et al. (2018). Endosomal rab cycles regulate Parkin-mediated mitophagy. eLife *7*, e31326.

Zhao, P., Wong, K.I., Sun, X., Reilly, S.M., Uhm, M., Liao, Z., Skorobogatko, Y., and Saltiel, A.R. (2018). TBK1 at the crossroads of inflammation and energy homeostasis in adipose tissue. Cell *172*, 731–743.

STAR★METHODS

KEY RESOURCES TABLE

REAGENT or RESOURCE	SOURCE	IDENTIFIER
Antibodies		
Actin	CST (Cell Signaling Technology)	4967S
AMPK alpha1/2	CST	2532S; RRID:AB_10694064
ATG13	MBL	M183-3
ATG14	Proteintech	19491-1-AP; RRID:AB_10642701
Catalase	Abcam	ab16731
COXII	Abcam	ab110258; RRID:AB_10887758
FIP200	Proteintech	17250-1-AP; RRID:AB_10666428
Flag	Sigma Aldrich	F3165
Gapdh	Sigma-Aldrich	G9545; RRID:AB_796208
GFP	DSHB	GFP-1D2
HA	CST	3724S
LC3B	CST	2775S; RRID:AB_915950
Myc	CST	9405s
NDP52	CST	D1E4A ;RRID:AB_2732810
NDP52	Sigma Aldrich	SAB1406957; RRID:AB_10741310
p62	Abnova	H00008878-M01; RRID:AB_437085
p70S6K	CST	2708P
p70S6K T389	CST	9205
Parkin	Santa Cruz	PRK8; RRID:AB_628104
TBK1	CST	D1B4; RRID:AB_2255663
TBK1 S172	CST	5483S; RRID:AB_10693472
TBK1 S172	Abcam	EPR2867(2)
Tim23	Santa Cruz	T-20
Tom20	Santa Cruz	FL145 (Rabbit) / F-10 (Mouse)
ULK1	CST	D8H5; RRID:AB_11178668
ULK1 pS757	CST	6888S
Alexa Fluor 405 goat anti-rabbit IgG (H+L)	Thermofisher	A31556
Alexa Fluor 488 goat anti-rabbit IgG (H+L)	Thermofisher	A11008
Alexa Fluor 546 goat anti-rabbit IgG (H+L)	Thermofisher	A11035
Alexa Fluor 594 goat anti-rabbit IgG (H+L)	Thermofisher	A11012
Alexa Fluor 633 goat anti-rabbit IgG (H+L)	Thermofisher	A21071
Alexa Fluor 647 goat anti-rabbit IgG (H+L)	Thermofisher	A21244
Alexa Fluor 488 goat anti-mouse IgG (H+L)	Thermofisher	A21202
Alexa Fluor 546 goat anti-mouse IgG (H+L)	Thermofisher	A11003
Alexa Fluor 594 goat anti-mouse IgG (H+L)	Thermofisher	A11005
Alexa Fluor 633 goat anti-mouse IgG (H+L)	Thermofisher	A21052
Alexa Fluor 647 goat anti-mouse IgG (H+L)	Thermofisher	A21235
Chemicals, Peptides, and Recombinant Proteins		
A/C heterodimerizer (Rapalog)	Takara	635057
antimycin A	Sigma-Aldrich	A8674
Bafilomycin	Sigma-Aldrich	B1793
DAPI	Thermofisher	62248
DRAQ7	CST	7406S

(*Continued on next page*)

Continued

REAGENT or RESOURCE	SOURCE	IDENTIFIER
DSP(dithiobis(succinimidyl propionate))	Thermofisher	22585
MRT67307, TBK1 inhibitor	Selleckchem	S7948
MRT68921, ULK1 inhibitor	Selleckchem	S7949
Oligomycin	CalBiochem	495455
Polybrene	Sigma-Aldrich	H9268
Q-VD	ApexBio	A1901
Valinomycin	Sigma-Aldrich	V0627
FuGENE HD	Promega	E231A
Omni-Avalance	EZ Biosystem	EZT-OMNI-1
DMEM	Thermofisher	31053028
McCoy's 5A	Thermofisher	16600082
Sodium pyruvate	Thermofisher	11360070
GlutaMax	Thermofisher	35050061
Nonessential amino acids	Thermofisher	11140050
HEPES	Thermofisher	15630080
Opti-MEM	Thermofisher	31985062
0.05% Trypsin-EDTA	Thermofisher	25300054
BamBanker	Wako	302-14681
DTT	Sigma-Aldich	D0632
HBSS with calcium and magnesium	Thermofisher	14025-092
paraformaldehyde 16% solution, EM grade	Electron Microscopy Sciences	15710
polyethylenimine, linear, MW 25000, transfection grade, (PEI 25K)	PolySciences	23966-1
PhosSTOP	Roche	4906845001
Protease inhibitor cocktail	Roche	11873580001
puromycin	Invivogen	ant-pr-1
Experimental Models: Cell Lines		
U2OS WT	Toyama et al., 2016	N/A
AMPK DKO U2OS	Toyama et al., 2016	N/A
FIP200 KO HeLa	This study	N/A
TBK1 KO HeLa	Lazarou et al., 2015	N/A
OPTN/NDP52/Taxbp1 TKO HeLa	Lazarou et al., 2015	N/A
Pink1 KO HeLa	Kane et al., 2014	N/A
ATG8 6KO HeLa	Nguyen et al., 2016	N/A
OPTN/NDP52/Taxbp1/p62/NBR1 5KO HeLa	Lazarou et al., 2015	N/A
HeLa	ATCC	CCL-2.2
293T	ATCC	CRL-3216
HCT116	ATCC	CCL-247
TBK1 KO HCT116	This study; CRISPR sgRNA used in Lazarou et al., 2015	N/A
mt-mKeima/YFP-Parkin stable HeLa	Lazarou et al., 2015	N/A
mCherry-Parkin stable HeLa	Lazarou et al., 2015	N/A
Oligonucleotides		
FIP200-e4-5′ crispr	GCTATGTAAAAACACCTTAG	N/A
FIP200-e4-3′ crispr	GTAGTTTTAGGAATAGCAGG	N/A
FIP200-e4-CEL1-F	AGACCTGATAACCAGTTTGAGCAT	PCR product 799 bp. 435 bp deletion with CRISPR
FIP200-e4-CEL1-R	TGTCAAACTTTTTGCATACTTCCT	N/A

(*Continued on next page*)

Continued

REAGENT or RESOURCE	SOURCE	IDENTIFIER
Recombinant DNA		
p3xFLAG-CMV-hATG13	Addgene	22872
p3xFLAG-CMV10-hFIP200	Addgene	24300
p3XFLAG-CMV10-mApg16L	Addgene	24302
pBMN-FKBP-mEGFP-ATG14	This study	N/A
pBMN-FKBP-mEGFP-ATG16L1	This study	N/A
pBMN-FKBP-mEGFP-ATG16L1FBD	This study	N/A
pBMN-FKBP-mEGFP-ATG16L1FBD_E230R_E241R	This study	N/A
pBMN-FKBP-mEGFP-mULK1	This study	N/A
pBMN-FKBP-mEGFP-mULK1_T180A	This study	N/A
pBMN-FKBP-mEGFP-NDP52	This study	N/A
pBMN-FKBP-mEGFP-NDP52_C425A	This study	N/A
pBMN-FKBP-mEGFP-TBK1	This study	N/A
pBMN-FKBP-mEGFP-TBK1_K38M	This study	N/A
pBMN-mCherry-Parkin	Lazarou et al., 2015	N/A
pBMN-mCherry-Parkin	Lazarou et al., 2015	N/A
pBMN-mEGFP-NDP52	Lazarou et al., 2015	N/A
pBMN-puro-P2A-FRB-Fis1	Lazarou et al., 2015	N/A
pBMN-YFP-Parkin	Lazarou et al., 2015	N/A
pC4-RhE-PMP34-FRB	Lazarou et al., 2015	N/A
pcDNA3-Flag-ULK1	Addgene	27636
pcDNA6.2-Myc-ULK1	Addgene	27629
pCHAC-mt-mKeima	Lazarou et al., 2015	N/A
pHAGE-FKBP-FLAG-HA-NDP52	This study	N/A
pHAGE-FKBP-FLAG-HA-TBK1	This study	N/A
pHAGE-FKBP-FLAG-HA-ULK1	This study	N/A
pHAGE-FKBP-GFP-ULK1	This study	N/A
pHAGE-FKBP-GFP-ULK1_S1042A	This study	N/A
pHAGE-FKBP-GFP-ULK1_S1047A	This study	N/A
pHAGE-FKBP-mEGFP-OPTN	This study	N/A
pHAGE-FKBP-mEGFP-TBK1	This study	N/A
pHAGE-FKBP-mEGFP-TBK1_dC	This study	N/A
pHAGE-FKBP-mEGFP-TBK1_dC-NDP52	This study	N/A
pHAGE-FKBP-mEGFP-TBK1_dC-NDP52_dSkich	This study	N/A
pHAGE-FKBP-mEGFP-TBK1_K38M	This study	N/A
pCR3-3XFlag-NDP52	Verlhac et al., 2015	N/A
pCR3-3XFlag-NDP52 dSKICH-dCLIR	Verlhac et al., 2015	N/A
pCR3-3XFlag-NDP52 C425A	Verlhac et al., 2015	N/A
pHAGE-FLAG-HA-NDP52	Lazarou et al., 2015	N/A
pHAGE-FLAG-HA-NDP52	This study	N/A
pHAGE-FLAG-HA-NDP52_C425A	This study	N/A
pHAGE-FLAG-HA-NDP52_dSkich_dCLIR	This study	N/A
pHAGE-FLAG-HA-NDP52_dSkich_dCLIR_C425A	This study	N/A
pHAGE-FLAG-HA-NDP52_dSkich_dCLIR_LIR-null	This study	N/A
pHAGE-FLAG-HA-NDP52_LIR-like-null	This study	N/A
pHAGE-GFP-FBD_EE-Parkin	This study	N/A
pHAGE-GFP-FBD-Parkin	This study	N/A
pHAGE-GFP-FBD-Parkin_K211N	This study	N/A

(*Continued on next page*)

Continued		
REAGENT or RESOURCE	SOURCE	IDENTIFIER
pHAGE-mt-mKeima-P2A-FRB-Fis1	This study	N/A
pHAGE-mTagBFP2-NDP52	This study	N/A
pME18s-HA-hFIP200	Addgene	24303
pMEs-3xHA-FIP200_C1	This study	N/A
pMEs-3xHA-FIP200_C2	This study	N/A
pMEs-3xHA-FIP200_C3	This study	N/A
pMEs-3xHA-FIP200_LZ only	This study	N/A
pMEs-3xHA-FIP200_LZ7A	This study	N/A
pMEs-3xHA-FIP200_LZ7A only	This study	N/A
pMEs-3xHA-FIP200_N1	This study	N/A
pMEs-3xHA-FIP200_Q4A	This study	N/A
pMXs-IP-EGFP-hFIP200	Addgene	38192
pMXs-IP-EGFP-ULK1	Addgene	38193
pMXs-IP-Venus-mULK1	Addgene	58743
pMXs-puro-GFP-DFCP1	Addgene	38269
pMXs-puro HA-ATG14	Addgene	22877
pRK5-Myc-mTOR	Addgene	1861
Others		
Amersham ECL Prime WB detection reagent	GE Healthcare Life Sciences	RPN2232
SuperSignal West Femto Luminol/Enhancer solution	Thermofisher	1859022
SuperSignal West Femto stable peroxide buffer	Thermofisher	1859023
NEBuilderHiFi DNA assembly master mix	New England BioLabs	M5520A
Q5 Hot Start High-Fidelity DNA Polymerase	New England BioLabs	M0493L
Phusion Hot Start II DNA polymerase	Thermofisher	F-549L
DNA Ligation kit, Mighty Mix	Takara	6023
LDS sample buffer (4x)	Thermofisher	NP0007
GFP-TRAP magnetic beads	Chromotek	gtma-20
FLAG-beads	Sigma-Aldrich	M8823
Triton X-100	Sigma-Aldrich	T9284
Tween-20	Sigma-Aldrich	P7949
3xFLAG peptide	Thermofisher	A36806
Protein A/G magnetic beads	Thermofisher	88802
HA Beads	Thermofisher	N/A
Software and Algorithms		
MATLAB		N/A
ImageJ		N/A
Prism		N/A

CONTACT FOR REAGENT AND RESOURCE SHARING

Further information and requests for resources and reagents should be directed to and will be fulfilled by the Lead Contact, Richard Youle (youler@ninds.nih.gov)

EXPERIMENTAL MODEL AND SUBJECT DETAILS

Cell Lines

HEK293T, HeLa and HCT116 cells were purchased from ATCC. HEK293T, U2OS and HeLa cells were cultured in Dulbecco's modified eagle medium (DMEM) with 10% (v/v) Fetal Bovine Serum (FBS) (Gemini Bio and Sigma), 1 mM Sodium Pyruvate, 1x nonessential amino acids (NEAA), 2 mM GlutaMAX, and 10 mM Hepes. HCT116 cells were cultured in McCoy's 5A medium with 10% FBS, 2 mM

GlutaMax and 1x NEAA. WT and AMPK alpha ½ double KO U2OS (DKO, gifts from Dr. Reuben Shaw, Salk Institute, San Diego) were cultured in the same manner. All media and supplements were from Thermo Fisher. All cells were tested for mycoplasma contamination every two weeks with PlasmoTest kit (InvivoGen). Reagents used for transfections were Avalanche-OMNI (EZ Bio-Systems), X-tremeGENE 9 (Roche), FuGeneHD (Promega) or Polyethylenimine (PEI) (PolySciences). The full list of antibodies and reagents are found in Key Resources Table.

METHOD DETAILS

Knock out line generation using CRISPR/Cas9 gene editing

CRISPR gRNAs were generated to target common exons of FIP200 shared by all splice variants. Two gRNAs (ACTATGTAAAAA CACCTTAG and GTAGTTTTAGGAATAGCAGG) were used to delete the entire frame-shifting exon 4 and exon 5 of FIP200. gRNAs were cloned into pSpCas9(BB)-2A-Puro (PX459) V2.0 (Addgene plasmid #62988). To Make FIP200 KO, HeLa cells were transfected with the two gRNA plasmids and treated with 1 ug/ml puromycin for 2 days to enrich transfected cells, which were then diluted and placed into 96-well plates for single colonies. Primer set (AGACCTGATAACCAGTTTGAGCAT and TGTCAAACTTTTTGCA TACTTCCT) were used for PCR screening of FIP200 KO clones. TBK1 gRNA was previously reported (Lazarou et al., 2015) and used to make HCT116 TBK1 KO cells following similar protocol (Lazarou et al., 2015).

Cloning and stable cell line generation

For retroviral and lentiviral constructs, inserts were either amplified by PCR or synthesized (Thermo Fisher) and cloned into pBMN-Z vector or pHAGE vector, respectively by Gibson assembly (New England Labs) or Gateway cloning (Thermo Fisher). Deletion mutants were generated using Gibson Cloning. Point mutants were generated by site directed mutagenesis. All constructs used or generated in this study were validated by Sanger sequencing and complete plasmid sequences and maps are available upon request.

Stable expression of retroviral and lentiviral constructs in HeLa cells were achieved as follows: retroviruses and lentiviruses were packaged in HEK293T cells by transfecting constructs together with appropriate helper plasmids and PEI. The next day, fresh media were added. Viruses were harvested 48 hrs and 72 hrs after transfection and transduced in HeLa cells with 8 μg/mL polybrene (Sigma). Cells were then directly used in experiments or optimized for expression by FACS.

Western Analyses

Cells seeded into 6-well or 12-well plates were washed with phosphate buffered saline (PBS) and lysed with LDS sample buffer (Thermo Fisher) with 50 mM dithiothreitol (DTT, Sigma). Samples were boiled at 99°C for 10 min. 20-50 μg of protein lysate of each sample was loaded and separated on 4-12% Bis-Tris gels (Thermo Fisher) according to manufacturer's protocol. Gels were transferred to polyvinyl difluoride membranes and immunostained using specific antibodies. For mitophagy measurements by immunoblotting, cells were treated with 10 μM Oligomycin (Calbiochem), 10 μM Antimycin A (Sigma) and 10 μM QVD (ApexBio) in growth medium at different timepoints indicated in figure legends, prior to western blot analysis. For mitophagy measurements after ectopic protein localization to mitochondria, cells were treated with A/C heterodimerizer Rapalog (Clonetech) for various periods, indicated in figure legends, prior to western blot analysis.

Immunoprecipitation, GFP-TRAP, HA and Flag beads precipitation

For GFP-TRAP (Chromotek), HA-beads (Pierce), Flag-beads (Sigma-Aldrich) precipitation experiments, HEK293T cells seeded in 6-well plates were co-transfected with specific constructs to overexpress proteins of interest for 24 to 48 h. Cells were then lysed using ice cold lysis buffer (150 mM Tris-buffered Saline, 50 mM NaCl, with 0.5% v/v Triton-X 100) supplemented with EDTA-free cOmplete protease inhibitor (Roche). Samples were incubated on ice with intermittent agitation by pipetting for 30 min. Beads were equilibrated using bead resuspension buffer (150 mM Tris-buffered Saline, 50 mM NaCl, 0.25% Tween). Protein lysates were precleared by centrifugation at 4°C for 10 min at 15,000 g. Clarified lysates were incubated with specific equilibrated beads for various periods at 4°C. Beads were then washed with ice cold Wash buffer (150 mM Tris-buffered Saline, 50 mM NaCl) 3 to 5 times. Bound proteins were eluted with LDS lysis buffer (Thermo Fisher) with 50 mM DTT. GFP-TRAP proteins were eluted in boiling conditions while HA-bead bound proteins were eluted at 37°C for 10 min. Flag-bead bound proteins were eluted by competition using 3X Flag (Pierce) as recommended by the manufacturer. Beads were then separated from eluent. GFP-TRAP eluent samples were boiled for an additional 10 min. Samples were processed for immunoblotting as described in the western blot analyses section.

For endogenous immunoprecipitation, cells were lysed in lysis buffer and clarified, as shown above. Clarified lysates were first incubated with antibodies specified in figure legends for 24 h at 4°C. A/G beads (Pierce) were equilibrated with resuspension buffer then incubated with antibody-lysate mix for 1 h at 4°C. Beads were washed as above. Bound samples were eluted using LDS lysis buffer with 50 μM DTT at 37°C. Samples were processed for immunoblotting as above. However, to reduce denatured IgG background bands, TrueBlot (Rockland) secondary antibodies were used. In some experiments, 200 nM of Bafilomycin (Selleckchem) was used for various periods.

Immunofluorescence microscopy

Cells seeded in 2-well or 4-well chamber slides (Lab-Tek) were treated as indicated in figure legends. After treatment, cells were rinsed in PBS and fixed with 4% paraformaldehyde at RT for 10 min. Cells were washed with PBS. For immunostaining, cells were then permeabilzed and blocked with 0.1% Triton X-100, 3% goat serum in TBS or PBS for 15 min at RT. After, cells were incubated with 0.1% Triton X-100, 3% goat serum in TBS or PBS supplemented with antibodies (1:1000) overnight at 4°C. Cells were then washed with PBS 3 times and incubated with Alexa 488 or 546, 633-conjugated secondary antibodies (Thermo Fisher). For cells expressing fluorescent tagged proteins, cells were seeded as above. After treatments, cells were fixed as above and washed 3 times with PBS prior to image analysis. For live imaging of mito-mKeima of cells in Figure 1B, live cells in 2-chamber slides were treated with Rapalog for 24 h, placed in a CO2 chamber platform of LSM 880 at 37°C. Images were taken using a 20x air, 63x oil, or 40x oil DIC objective on an LSM 510 or 880 microscope (Zeiss).

Image Analysis

To quantify colocalization, Pearson's correlation coefficients were quantified with a custom MATLAB script. Briefly, images were background subtracted then cells segmented from background by intensity and size prior to pixel-by-pixel correlation. Tile images were used to increase the number of cells quantified, then different sites of the chamber slide were used to find the average and error for the experiment. P values were determined with an unpaired Student's T-test from different sites. We also generated custom MATLAB scripts for automated puncta counting. The images were background subtracted then several binary masks created to find puncta of interest including a mask for cells positive for transfection, a mask for mitochondria using a TOM20 stain, a mask for FKBP-GFP positive subcellular location, and a mask for puncta. The puncta mask was made by passing the background subtracted image through Gaussian filters then dividing the image by those filters to accentuate regions of high contrast. Two-dimensional maxima were found across the image, filtered by size, then filtered by all masks to find legitimate protein puncta amid noise and highly transfected cells. Puncta were then counted and divided by the number of positive cells, distinguished and segmented with a nuclear dye.

Mitochondrial recruitment of proteins during Parkin-mediated mitophagy

Cells stably expressing or transiently expressing constructs, as specified in figure legends were seeded in 2-well or 4-well chambers (Lab-Tek). Cells were treated with 10 μM Oligomycin (Calbiochem), 10 μM Antimycin A (Sigma) and 20 μM QVD (ApexBio). Cells were simultaneously treated with 200 nM Bafilomycin to prevent degradation of proteins of receptor and ATG proteins.

Chemically inducible dimerization assays

For mitochondrial localization of NDP52, ULK1, FIP200-binding peptide (FBD) and TBK1, 2XFKBP was fused to GFP-Tagged NDP52, ULK1, FBD, or TBK1 in pBMN retroviral or pHAGE lentiviral vector. For mitochondrial recruitment assays, cells stably expressing specific FKBP construct with FRB-Fis1 and mito-mKeima (previously described by Lazarou et al., 2015) were treated with 0.5 μM A/C heterodimerizer (simply named as Rapalog in the text) (Clontech) for 24 h. Cells were either imaged live or fixed and immunostained as described above.

Mitophagy assay with mito-mKeima via fluorescence activated cytometer

Stable cell lines were generated to express mito-mKeima, FRB-Fis1 or FRB-mTagBFP2-Fis1, and FKBP-GFP-tagged target genes with retrovirus or lentivirus system. 100K cells were seeded in 12-well plates and treated with Rapalog for 24h or by OA at various time points before FACS analysis. FACS analysis were performed as previously described (Lazarou et al., 2015).

Pexophagy assay

About 50-100K HeLa cells were seeded in 2-well chamber slides (Labtek) and transfected with 0.2 ug of mito-GFP, 0.6 ug of FRB-PMP34 and 0.4 ug of FKBP-FLAG-HA-NDP52 or 0.8 ug of FKBP-FLAG-HA-TBK1 with 1:3 ratio of FuGeneHD (Progemga). 18hr after transfection, cells were treated with Rapalog for 30h or 48h, fixed and immunostained with anti-HA (Cell Signaling) and anti-catalase (CalBiochem).

QUANTIFICATION AND STATISTICAL ANALYSIS

All statistical analyses for FACS were calculated in GraphPad Prism 6. Statistical significance was assessed from three independent experiments using one-way or two-way ANOVA with a confidence interval of 95%. Error bars are expressed as mean ± standard deviation.

Molecular Cell, Volume *74*

Supplemental Information

Spatiotemporal Control of ULK1 Activation

by NDP52 and TBK1 during Selective Autophagy

Jose Norberto S. Vargas, Chunxin Wang, Eric Bunker, Ling Hao, Dragan Maric, Giampietro Schiavo, Felix Randow, and Richard J. Youle

SUPPLEMENTAL FIGURES

Figure S1. NDP52 interacts with ULK1 complex through FIP200, related to Figures 1, 2 and 3.

(A) Western analysis of GFP-TRAP from HeLa cells stably expressing mCherry-Parkin and GFP-NDP52. Cells were treated with OA and Bafilomycin (100 nM) for 2 h. (B) Western analysis of NDP52 levels in WT or FIP200 KO HeLa cells.
(C) Endogenous immunoprecipitation of ULK1 in WT HeLa or FIP200 KO cells. (D) Western analysis of GFP-TRAP from HEK293T cells transiently expressing GFP-ULK1, FLAG-NDP52, and HA-FIP200. (E) Confocal imaging of HeLa cells stably expressing mito-mKeima, FRB-Fis1 and FKBP-GFP-NDP52 WT or ΔSKICH mutant. Cells were transfected with HA-FIP200, followed by 24 h treatment of Rapalog. Cells were then immunostained for HA and Tom20. Arrows indicate triple colocalization of FKBP-GFP-NDP52, FIP200, and Tom20. Scale bars: 10 μm

Figure S2. Mitochondrial recruitment of NDP52 in the absence of TBK1, related to Figures 4 and 6.

(A) Confocal images of WT or TBK1 KO HeLa cells stably expressing BFP-NDP52 and mCherry-Parkin. Cells were treated with OA and Bafilomycin (200 nM) for 3 h. (B-C) Quantification of Pearson’s correlation coefficient between NDP52 and Parkin with or

without OA treatment for 3 or 24 h. (D) Confocal imaging of WT HeLa cells expressing various BFP-NDP52 mutants after 3 h treatment with OA (See Fig. 3A). (E) Western analysis of HA-Ub immunoprecipitation. HEK293T cells transiently expressing HA-Ubiquitin with FLAG-NDP52, FLAG-NDP52-ΔSKICH-ΔCLIR, or Flag-NDP52 (C425A). Red arrows indicate NDP52 that is bound to ubiquitin. Data are represented as mean ± SD. p-value: * = < 0.05; ** < 0.01; *** < 0.001; **** < 0.00001; ns., not significant. Scale bars: 10 μm

Figure S3. Ectopic mitochondrial localization of ATG16L1 FIP200-binding domain can initiate mitophagy via FIP200, related to Figures 5, 6 and 7.

(A) Diagram of FIP200 binding domain (FBD) from ATG16L1 fused with FKBP. (B) Quantification of FACS analysis for mito-mKeima. Cells stably expressing mito-mKeima, FRB-Fis1, and FKBP-GFP-FBD or FKBP-GFP-FBD (E230R/E241R) mutant were treated with Rapalog for 24 h. (C) Western analysis of GFP-TRAP assay performed on HEK293T cells overexpressing FKBP-GFP-FBD and (E230R/E241R) mutant. (D) Quantification of FACS ratiometric analysis of mito-mKeima. Cells stably expressing mito-mKeima, FRB-Fis1, and FKBP-GFP-FBD were treated with Rapalog alone or with co-treatment of ULK1/2 inhibitor (MRT68921 1 μM) or TBK1 inhibitor (MRT67307 2 μM) for 24 h. (E) Quantification of FACS ratiometric analysis of mito-mKeima. WT or AMPK alpha 1/2 double KO (DKO) stably expressing mito-mKeima, FRB-Fis1, and FKBP-GFP-FBD. Cells were treated with Rapalog for 24 h. All FACS quantifications: n = 3

biological replicates. Data are represented as mean ± SD. p-value: * = < 0.05; ** < 0.01; *** < 0.001; **** < 0.00001; ns., not significant.

Figure S4. LC3 lipidation defect in the absence of AMPK in human cells and effect of ULK1 autophosphorylation sites, related to Figure 5.

(A) Western analysis of WT or FIP200 KO HeLa cells after starvation with HBSS with or without Bafilomycin. (B-C) Western analysis. WT U2OS and AMPK DKO were starved for 4 h (B) or 24h (C) with or without Bafilomycin. (D) Cells in (B) were treated with Torin1 for 4h with or without Bafilomycin. (E) FACS ratiometric mito-mKeima analysis. HeLa cells expressing FKBP-GFP-ULK1 WT, S1042A, or S1047A, with FRB-Fis1, and mito-mKeima were treated with Rapalog for 24 h. All FACS quantifications: n = 3 biological replicates. Data are represented as mean ± SD. p-value: * = < 0.05; ** < 0.01; *** < 0.001; **** < 0.00001; ns., not significant

Figure S5. TBK1 positively regulates mitophagy rate with NDP52, related to Figure 6

(A) Western analysis of COXII and Tim23 to measure mitophagy in WT or TBK1 KO HeLa cells. Cells stably expressing mCherry-Parkin were treated with OA for 6, 18, or 24 h. (B) Western analysis of mitophagy in WT or TBK1 KO HCT116 cells. Cells stably expressing mCherry-Parkin were treated with OA for 6, 18 and 24 h. (C) Western

analysis of HCT116 after treatment of OA with or without Bafilomycin for 6 or 16h. (D) Western analysis of ULK1 complex levels in WT or TBK1 KO HeLa cells. (E) Schematics of chimeric NDP52-TBK1 generated for mitophagy rescue analysis in (F) and (G). (F-G) FACS ratiometric analysis of mito-mKeima in (F) TBK1 KO cells or in (G) OPTN, NDP52, Tax1bp1 TKO cells expressing the various TBK1-NDP52 constructs. Cells were treated with OA for 4 h prior to FACS. All FACS quantifications: n = 3 biological replicates. Data are represented as mean ± SD. p-value: * = < 0.05; ** < 0.01; *** < 0.001; **** < 0.00001; ns., not significant

Figure S6. Autophosphorylation of TBK1 upon cargo localization, related to Figure 6.

(A-B) Confocal imaging of phosphorylated TBK1 S172. HeLa cells expressing FRB-Fis1, mito-mKeima, and FKBP-GFP-TBK1 (A) or K38M kinase-dead FKBP-GFP-TBK1 (B) were treated with Rapalog and QVD for 24h. Cells were then immunostained for Tom20 and TBK1 S172 prior to imaging. (C-D) Pearson's correlation coefficient between GFP-FKBP-TBK1 or K38M mutant and Tom20 (C) and between TBK1 phosphorylated at S172 and Tom20. (E) Western analysis of S172 phosphorylation. HeLa cells expressing FRB-Fis1, mito-mKeima, and FKBP-GFP-TBK1 or or K38M kinase-dead FKBP-GFP-TBK1 were treated with Rapalog for 24 h. Data are represented as mean ± SD. p-value: * = < 0.05; ** < 0.01; *** < 0.001; **** < 0.00001; ns., not significant

Figure S7. Pexophagy induced by NDP52, ULK1, and TBK1 localization, related to Figure 5, 6, and 7.

(A) Confocal imaging of catalase staining in WT or 6KO (LC3/GABARAP hexa KO) HeLa cells. Cells are transiently expressing FKBP-FLAG/HA-NDP52, mito-GFP, and FRB-PMP34 and treated with Rapalog for 24 h. Cells were then fixed and stained for catalase and HA to ensure expression of FKBP construct (not shown). White asterisks denote cytosolic catalase staining while blue asterisks show aggregated catalase staining. (B) Quantification of catalase staining morphology in (A). (C) Quantification of peroxisome morphology in WT, 5KO (OPTN, NDP52, Tax1bp1, p62, NBR1 penta KO), and 6KO cells. Cells are transiently expressing FKBP/FLAG-HA-ULK1, mito-GFP, and FRB-PMP34. (D) Confocal imaging of catalase staining in WT or 5KO HeLa cells transiently expressing FKBP-FLAG/HA-TBK1, mito-GFP, and FRB-PMP34. Cells were treated with Rapalog for 24 h, fixed, then stained for catalase and HA (not shown). (E) Quantification of cells with either normal, cytosolic, or aggregated catalase staining in (D). (F) Quantification of catalase staining morphology in cells expressing FLAG/HA-NDP52 in WT or FIP200 KO cells. For all imaging experiments: Mito-GFP is used to track transfected cells, n = 3 biological replicates with at least 150 cells per condition counted in each experiment. Data are represented as mean ± SD. p-value: * = < 0.05; ** < 0.01; *** < 0.001; **** < 0.00001; ns., not significant.

Fig S1. NDP52 interacts with ULK1 complex through FIP200

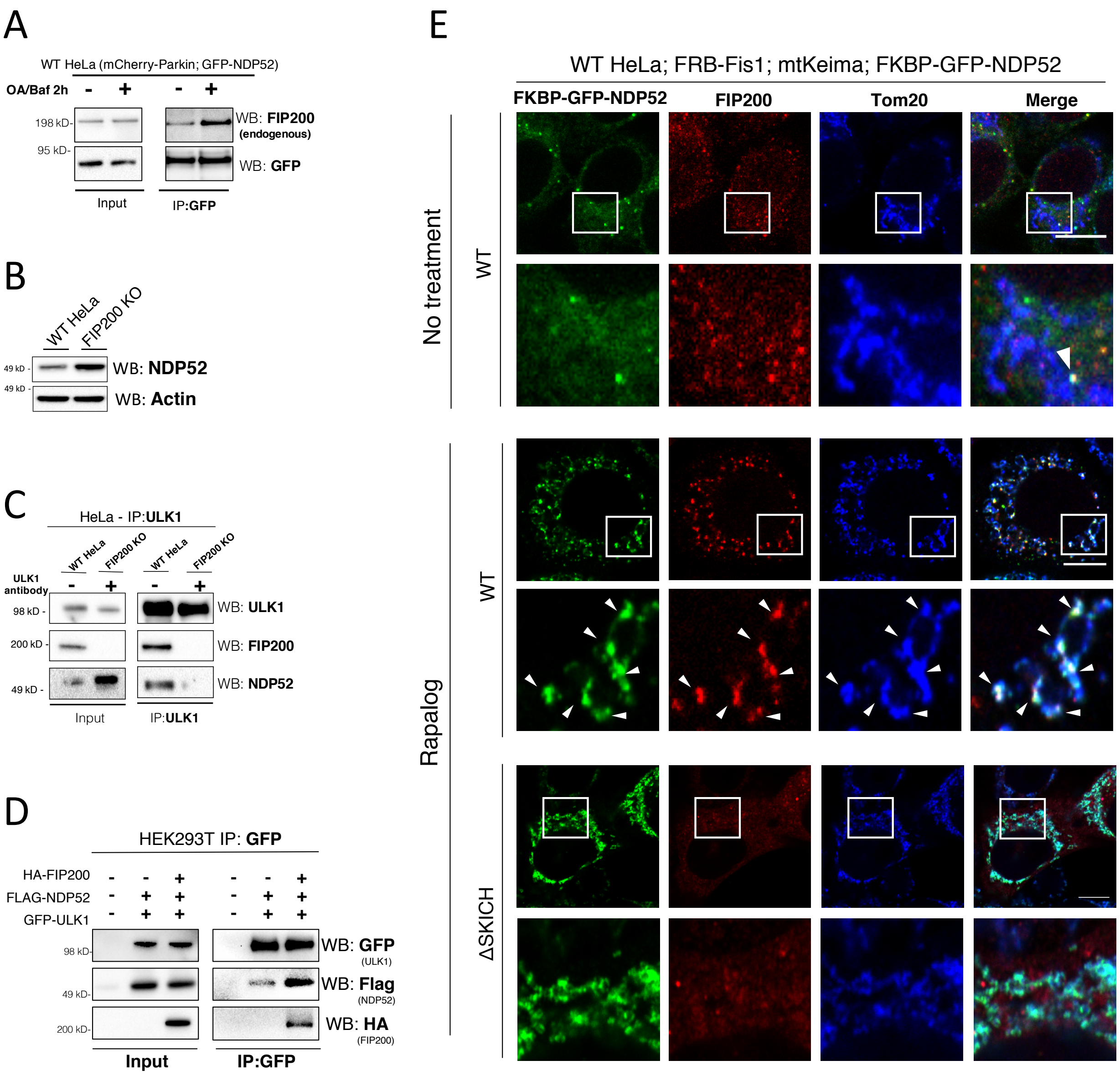

Fig S2. Mitochondrial recruitment of NDP52 in the absence of TBK1

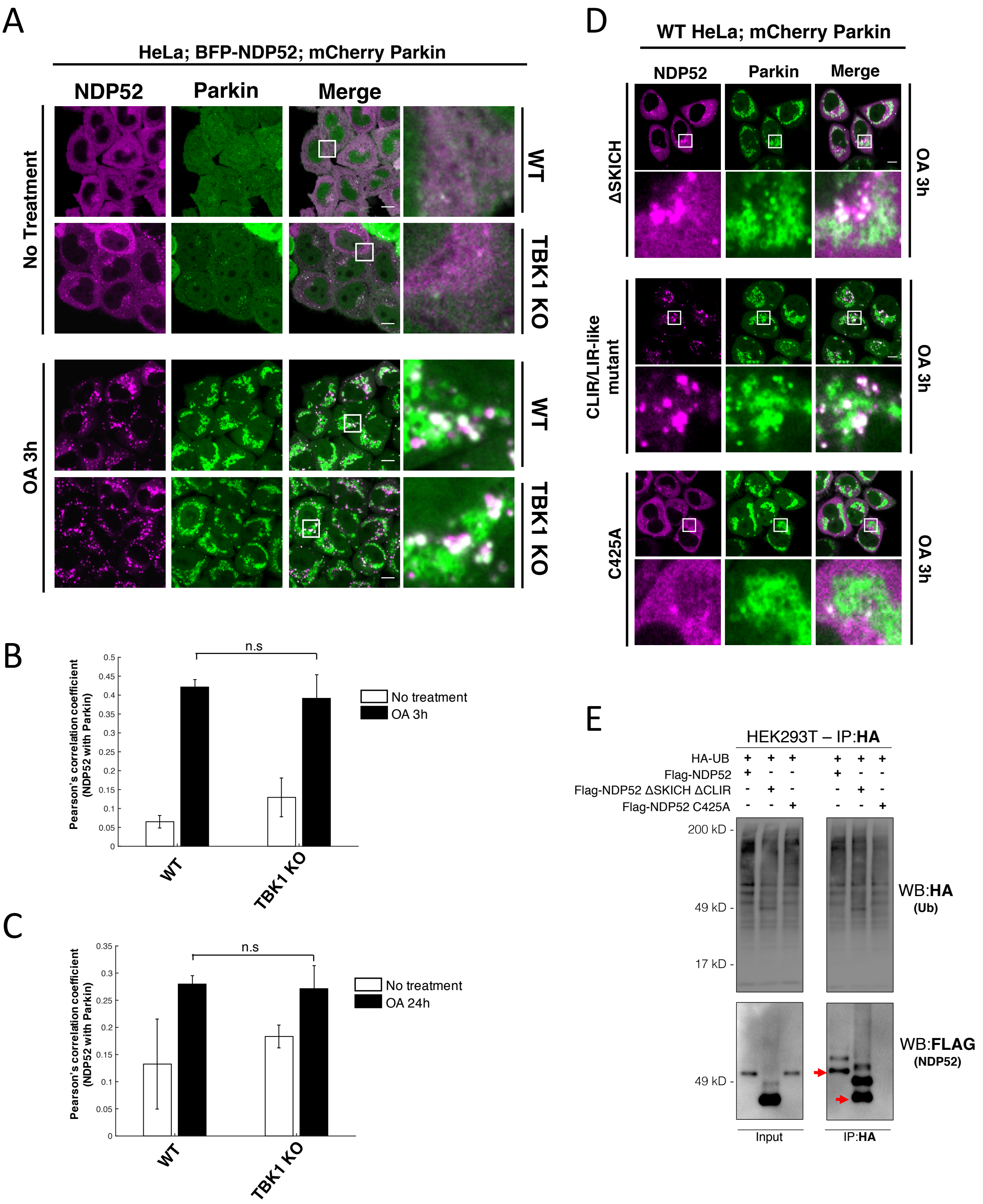

Fig. S3 Ectopic mitochondrial localization of ATG16L1 FBD domain can initiate mitophagy via FIP200

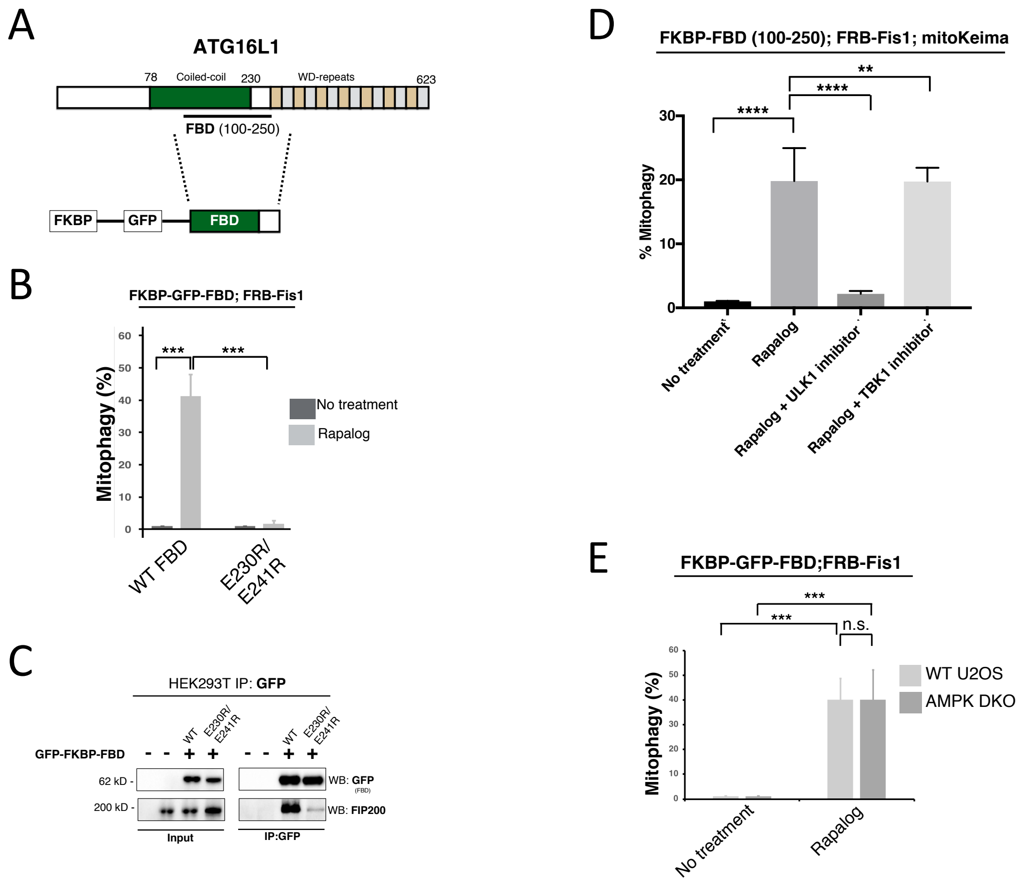

Fig. S4 LC3 lipidation defect in the absence of AMPK in human cells and effect of ULK1 autophosphorylation sites

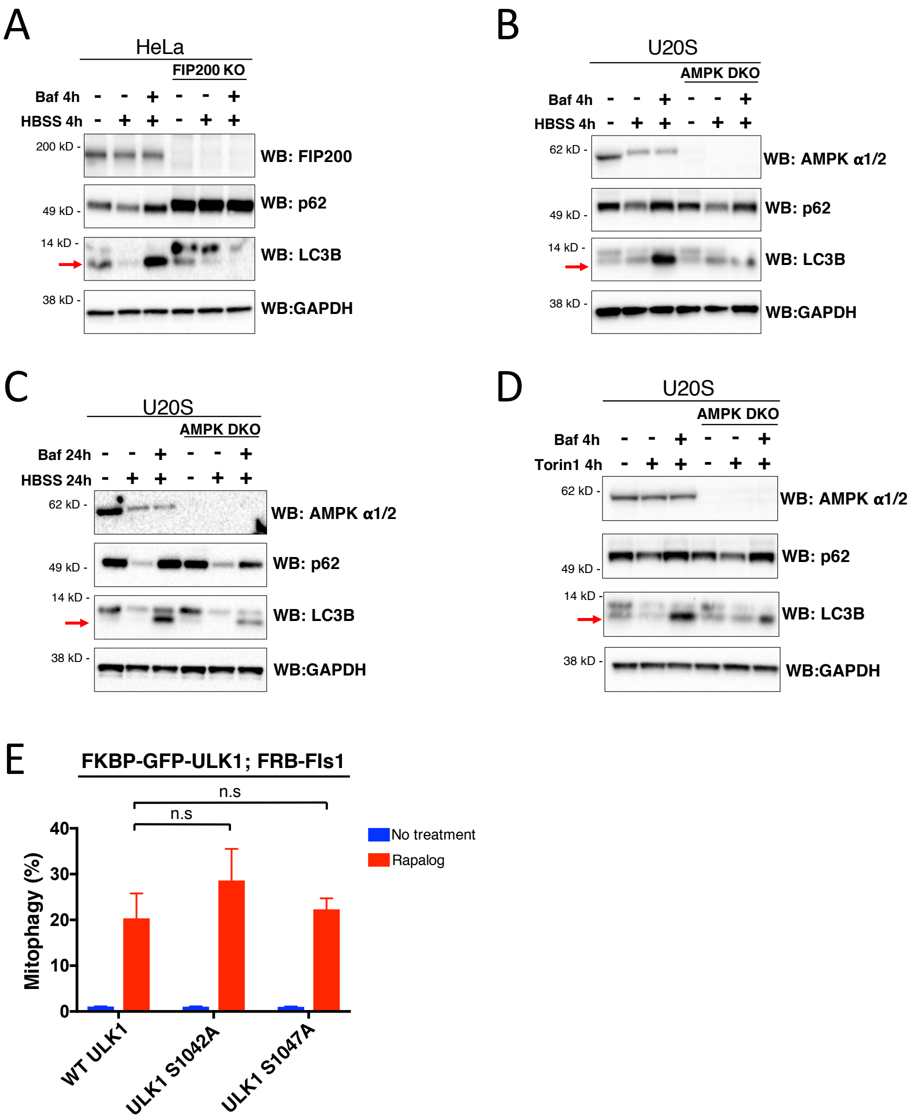

Fig. S5 TBK1 functions with NDP52 to positively regulate mitophagy rate

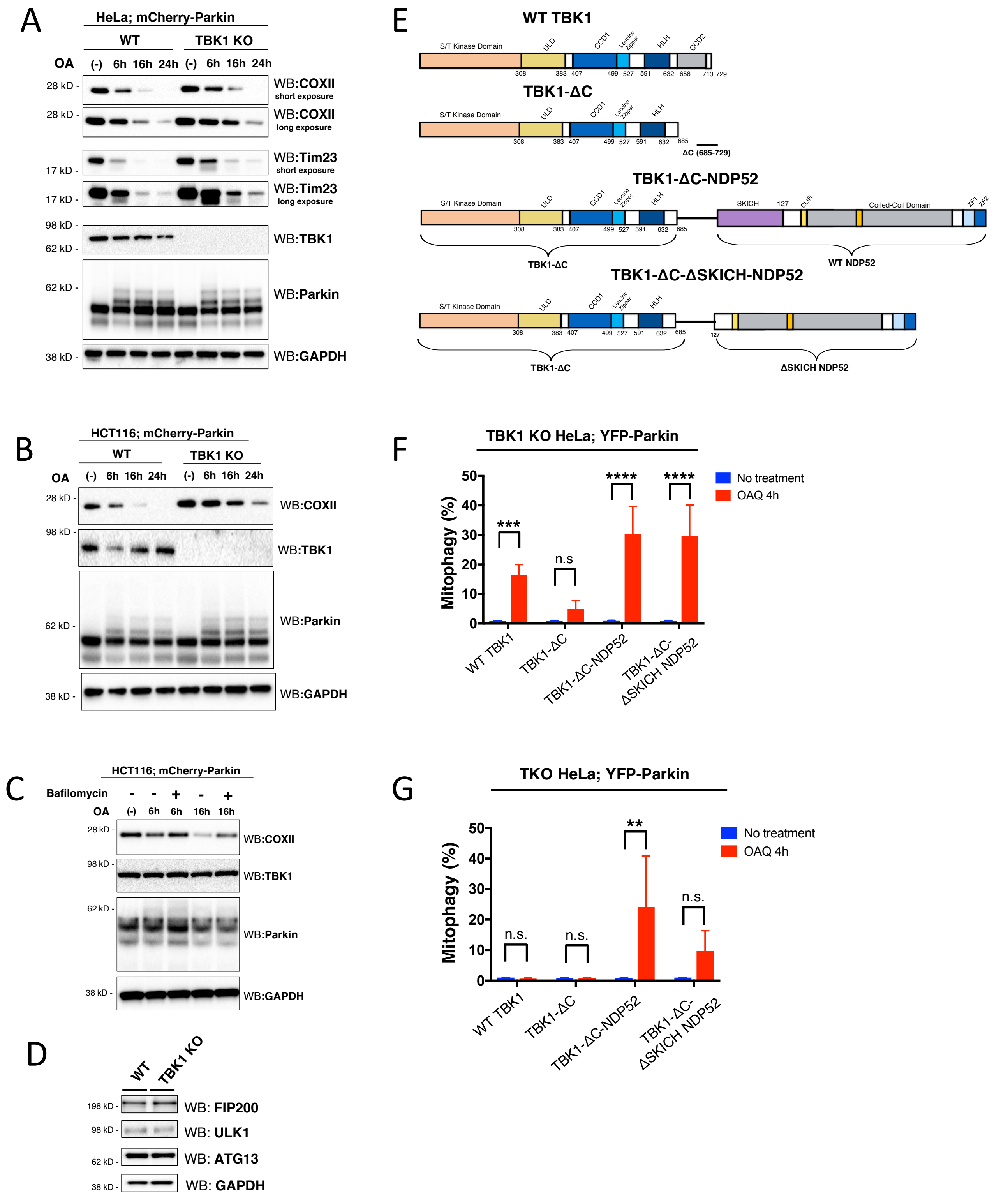

Fig. S6 TBK1 autophosphorylation on cargo

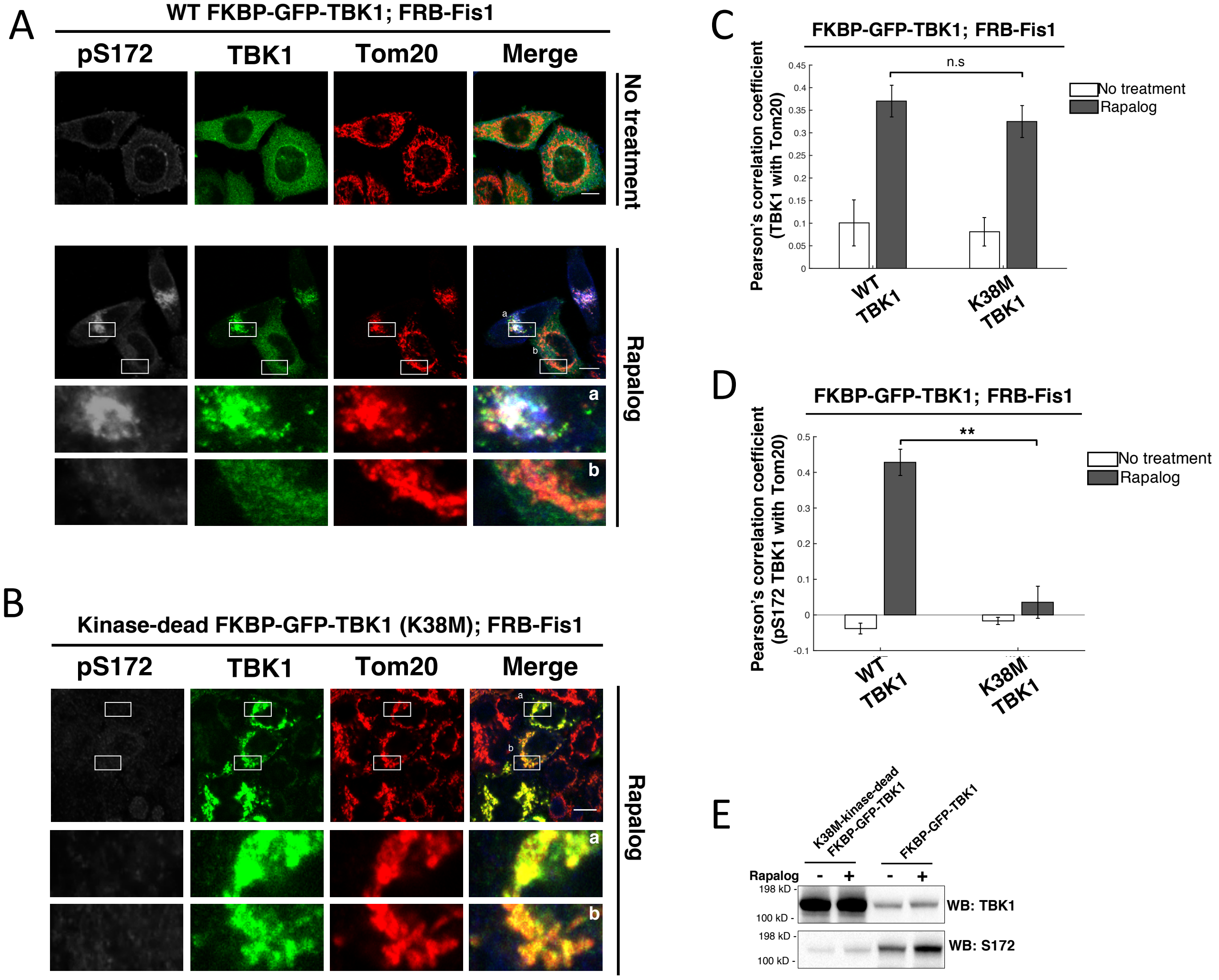

Fig S7 Pexophagy induced by NDP52, ULK1 and TBK1 localization

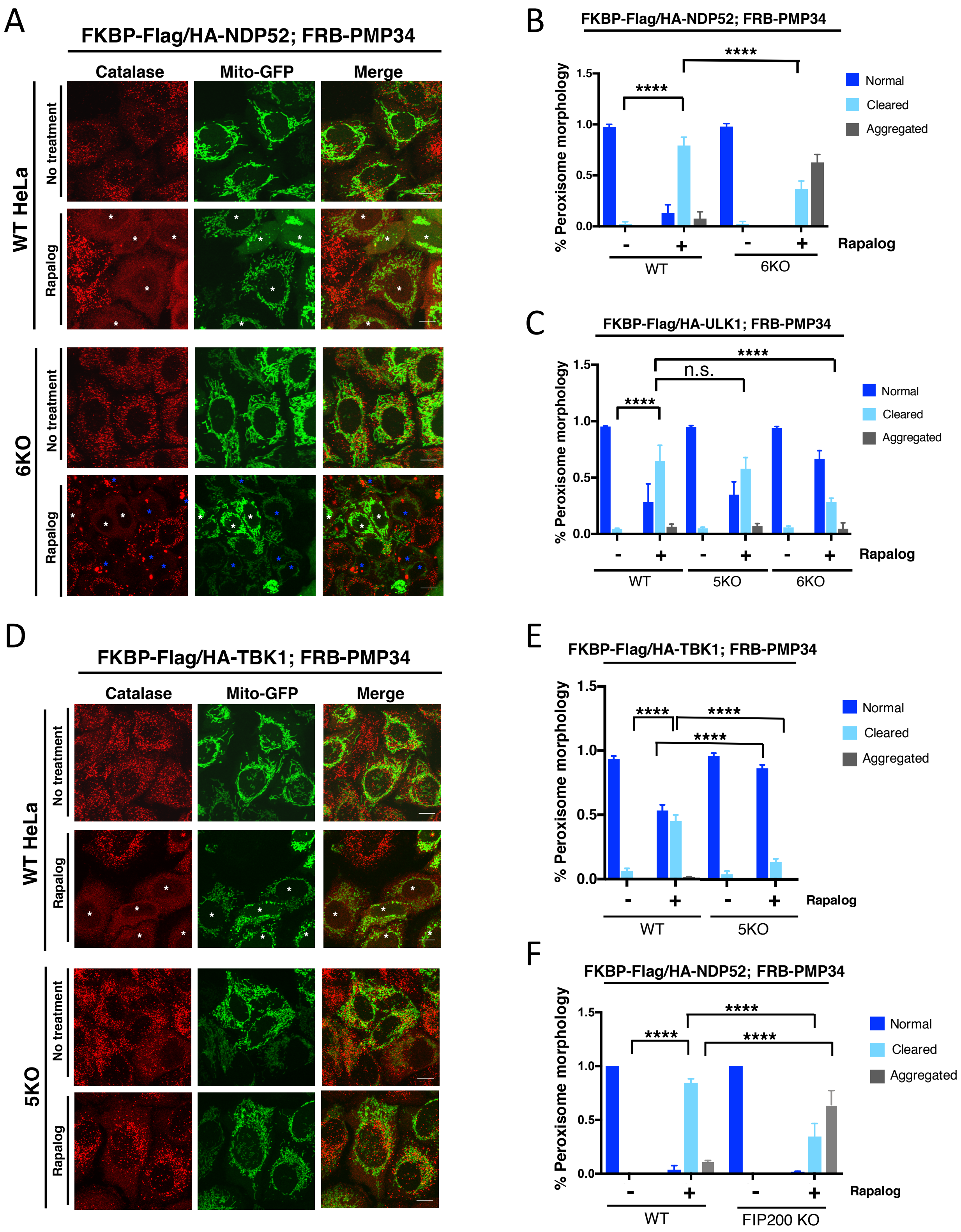

Supplement: Document S2. Article plus Supplemental Information [file mmc2.pdf]
